# Microglia Process α‐Synuclein Fibrils and Enhance their Pathogenicity in a TREM2‐Dependent Manner

**DOI:** 10.1002/advs.202413451

**Published:** 2024-12-12

**Authors:** Min Xiong, Danhao Xia, Honglu Yu, Lanxia Meng, Xingyu Zhang, Jiehui Chen, Ye Tian, Xin Yuan, Xuan Niu, Shuke Nie, Zhaohui Zhang, Chaoyang Liu, Qiang Chen, Keqiang Ye, Zhentao Zhang

**Affiliations:** ^1^ Department of Neurology Renmin Hospital of Wuhan University Wuhan 430060 China; ^2^ Frontier Science Center for Immunology and Metabolism Medical Research Institute Wuhan University Wuhan 430071 China; ^3^ Faculty of Life and Health Sciences Shenzhen Institute of Advanced Technology Shenzhen 518035 China; ^4^ TaiKang Center for Life and Medical Sciences Wuhan University Wuhan 430000 China

**Keywords:** α‐synuclein, asparagine endopeptidase, microglia, Parkinson's disease, triggering receptor expressed on myeloid cells 2

## Abstract

Parkinson's disease (PD) is characterized by the deposition of misfolded α‐synuclein (α‐syn) in the brain. Converging evidence indicates that the intracellular transmission and subsequent templated amplification of α‐syn are involved in the onset and progression of PD. However, the molecular mechanisms underlying the cell‐to‐cell transmission of pathological α‐syn remain poorly understood. Microglia is highly activated in the brains of PD patients. Here, it is shown that depletion of microglia slows the spread of pathological α‐syn pathology in mice injected with α‐syn fibrils. Microglia phagocytose α‐syn fibrils and transform them into more toxic species. The phagocytosis of α‐syn fibrils by microglia is partially mediated by triggering a receptor expressed on myeloid cells 2 (TREM2), a transmembrane protein expressed on the surface of microglia. The endocytosed α‐syn fibrils are then cleaved by the lysosomal proteinase asparagine endopeptidase (AEP) to generate truncated α‐syn 1–103 fibrils with enhanced seeding activity. Knockout of TREM2 and AEP impedes the endocytosis and cleavage of α‐syn fibrils, respectively. The results demonstrate that TREM2‐mediated phagocytosis of α‐syn fibrils by microglia and subsequent AEP‐mediated cleavage of α‐syn fibrils contribute to the spread of α‐syn in the brain. Blocking either of these two steps attenuates the progression of α‐syn pathology.

## Graphical abstract

In this study, TREM2 is found to mediate the endocytosis of α‐syn fibrils into microglia. The endocytosed α‐syn fibrils are then cleaved by the asparagine endopeptidase to generate truncated α‐syn 1‐103 fibrils with enhanced seeding activity. Blocking either of these two steps attenuates the progression of α‐syn pathology.

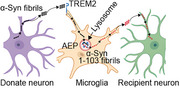

## 1 Introduction

Parkinson's disease (PD) is one of the most common neurodegenerative diseases.^[^ ^1^, ^2^ ^]^ The incidence of PD increases with age.^[^ ^3^, ^4^ ^]^ However, the etiology and pathogenesis of PD remain to be elucidated. Pathologically, PD is characterized by the progressive degeneration of dopaminergic neurons in the substantia nigra pars compacta (SNpc) and the formation of Lewy bodies (LBs) and Lewy neurites (LNs) within the remaining neurons. The major proteinaceous component of LBs and LNs is the aggregated form of α‐synuclein (α‐syn).^[^ ^5^ ^]^ α‐Syn is an intrinsically disordered protein abundantly distributed in the presynaptic terminals of neurons. In the brains of PD patients, α‐syn forms fibrils with well‐ordered β‐sheets. The conformational transformation of α‐syn is regulated by posttranslational modifications, such as phosphorylation,^[^ ^6^, ^7^ ^]^ ubiquitination,^[^ ^8^, ^9^ ^]^ and C‐terminal truncation.^[^ ^10^, ^11^ ^]^ Accumulating evidence from both human studies and animal models supports the cell‐to‐cell transmission of pathological α‐syn in the brain. It is believed that α‐syn fibrils are secreted from donor neurons and transferred to recipient neurons, where they recruit native α‐syn and template the assembly of *de novo* β‐sheet structures.^[^ ^12^ ^]^ However, the molecular mechanisms mediating the cell‐to‐cell spread of α‐syn pathology are poorly understood. There is evidence that supports the transsynaptic transmission of α‐syn.^[^ ^13^ ^]^ However, non‐synaptic pathways may also contribute to the spread of α‐syn pathology.^[^ ^14^, ^15^ ^]^

Microglia are resident phagocytes in the central nervous system. They survey the brain parenchyma and phagocytose dying cells, debris, and protein aggregates. Microglia also regulates the inflammatory process by secreting anti‐inflammatory and pro‐inflammatory cytokines. Thus, they govern neuronal function and homeostasis in the brain. Microglia are overactivated in the brains of PD patients.^[^ ^16^, ^17^ ^]^ Previous studies have shown that pathological α‐syn activates microglia and enhances their phagocytic activity.^[^ ^18^, ^19^ ^]^ Since microglia are highly endocytic and exocytic, they may play a role in the spread of α‐syn. Microglia have been shown to mediate the propagation of tau aggregates in a mouse model of tauopathy.^[^ ^20^ ^]^ We hypothesized that microglia may facilitate α‐syn propagation between neurons by phagocytosing and exocytosing pathological α‐syn seeds.

Triggering receptor expressed on myeloid cells 2 (TREM2) is a type I transmembrane protein that is expressed mainly on the surface of microglia. It consists of an extracellular ectodomain, a transmembrane region, and a short cytosolic tail. The ectodomain of TREM2 directly interacts with the environment to regulate microglial function.^[^ ^21^, ^22^, ^23^, ^24^, ^25^ ^]^ TREM2 has been found to play a key role in the onset and progression of Alzheimer's disease (AD).^[^ ^26^, ^27^ ^]^ However, it remains unknown whether TREM2 plays a role in the pathogenesis of sporadic PD. In this report, we show that TREM2 interacts with pathological α‐syn and mediates its endocytosis by microglia, a process that is diminished in TREM2 knockout (KO) mice. Once α‐syn fibrils enter microglia, they are processed by the lysosomal protease asparagine endopeptidase (AEP) and generate α‐syn 1–103 fibrils, which manifest stronger seeding activity. Knockout of AEP blocks the cleavage process but does not affect the endocytosis of α‐syn fibrils. Thus, we identified a two‐step biological event that mediates the spread of α‐syn pathology: TREM2‐mediated endocytosis of α‐syn fibrils and subsequent cleavage by AEP. Blocking either step of this process alleviates α‐syn pathology.

## 2 Results

### 2.1 Depletion of Microglia Attenuates the Propagation of α‐syn Pathology In Vivo

**Figure 1:**
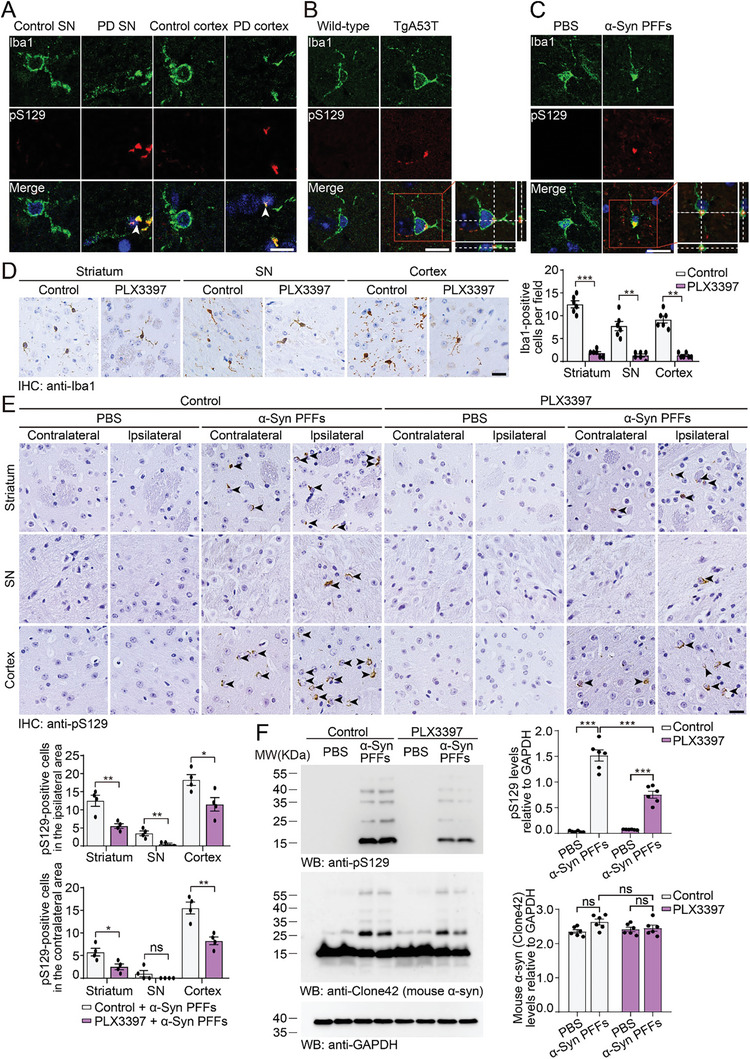
Depletion of microglia attenuates the propagation of α‐syn pathology in vivo. A–C) Immunofluorescence showing the colocalization of Iba1 and pS129 in the substantia nigra and cortex of brain sections from PD patients and age‐matched controls (A), TgA53T mice (B), and wild‐type mice injected with α‐syn PFFs (C). Iba1, green; pS129, red; DAPI, blue. Scale bar, 10 µm. D) Immunohistochemistry of Iba1 in the striatum, substantia nigra, and cortex after the mice were treated with PLX3397 for 1 month (mean ± s.e.m.; n = 6 mice per group; **p < 0.01; ***p < 0.001; *t*‐test and Mann‐Whitney test). Scale bar, 20 µm. E) Representative images of α‐syn pathology in mice treated with PLX3397 for 4 months (mean ± s.e.m.; n = 4 mice per group; *p < 0.05; **p < 0.01; *t*‐test and Mann‐Whitney test). Scale bar, 20 µm. F) Western blot analysis revealing the levels of pS129 and mouse α‐syn (Clone 42 antibody) in the striatum in mice treated with PLX3397 for 4 months (mean ± s.e.m.; n = 6 mice per group; ***p < 0.001; two‐way ANOVA). ns, not significant; SN, substantia nigra. See also in Figures S1–S4 (Supporting Information).

First, we performed immunofluorescence staining of brain sections from the post‐mortem substantia nigra and cortex of PD patients and age‐matched control subjects. We found that Iba1, a marker of microglia, partially colocalized with α‐syn phosphorylated at serine 129 (pS129), a marker of pathological α‐syn (**Figure** **1**A). Furthermore, pathological α‐syn was found to localize inside microglia in the striatum of α‐syn A53T transgenic mice (TgA53T mice, Line M83) and wild‐type mice that received intrastriatal injection of α‐syn pre‐formed fibrils (PFFs) (Figures 1B,C; Figure S1A–C, Supporting Information), suggesting that microglia phagocytose pathological α‐syn. To explore the potential role of microglia in the spread of α‐syn pathology, we depleted microglia by treating wild‐type mice with PLX3397 for one month and then unilaterally injected α‐syn PFFs into the striatum. PLX3397 treatment was continued throughout the experiment (Figure S1D, Supporting Information). PLX3397 is an inhibitor of colony‐stimulating factor 1 receptor (CSF1R), which specifically depletes microglia.^[^ ^28^ ^]^ One month after PLX3397 treatment, the density of Iba1‐positive microglia in the striatum, substantia nigra, and cortex was dramatically decreased, but the density of neurons and astrocytes was not affected after PLX3397 treatment (Figure 1D; Figure S2A,B, Supporting Information). Western blot analysis confirmed that the expression of the microglial marker P2Y12 was decreased in mice treated with PLX3397 for 1 month (Figure S2C, Supporting Information).

Intrastriatal injection of α‐syn PFFs into wild‐type mice triggers the spread of pathological α‐syn in the brain.^[^ ^29^ ^]^ To investigate the effect of microglial depletion on α‐syn pathology, we examined the extent of α‐syn pathology in the brains of mice treated with PLX3397 or vehicle at 1 month and 3 months after the injection of α‐syn PFFs. At 1 month post‐injection (mpi), pS129‐positive inclusions were found in the striatum, substantia nigra, and cortex ipsilateral to the injection site. The increase in α‐syn pathology was more pronounced in the PLX3397‐treated mice than in the control mice (Figure S1E, Supporting Information). At 3 mpi, α‐syn pathology was also detected in the striatum and cortex contralateral to the injection site. Interestingly, fewer pS129 signals were detected in the PLX3397‐treated mice than in the vehicle‐treated mice (Figure 1E). Western blot analysis revealed high‐molecular‐weight α‐syn species in the striatum of mice injected with α‐syn PFFs, which was partially attenuated by PLX3397 (Figure 1F). The levels of Iba1 and P2Y12 and the density of microglia in the ipsilateral striatum increased in the mice injected with α‐syn PFFs at 3 mpi and were partially attenuated by PLX3397 (Figure S3A,B, Supporting Information). Moreover, the mRNA levels of proinflammatory cytokines, including tumor necrosis factor‐α (TNF‐α), interleukin‐1β (IL‐1β), and IL‐6, in the ipsilateral striatum slightly increased at 1 mpi and dramatically increased at 3 mpi. The increase in pro‐inflammatory cytokines was partially alleviated in mice treated with PLX3397 (Figure S3C, Supporting Information). These results suggest that depletion of microglia partially attenuates the propagation of α‐syn pathology in vivo.

A panel of behavioral experiments was used to assess PD‐like motor deficits. In the tail suspension test, the mice injected with α‐syn PFFs exhibited clasping legs rather than spreading out both sides of their bodies, while these motor dysfunctions were partially ameliorated by PLX3397 treatment (Figure S3D, Supporting Information). In the rotarod test, grid performance test, pole test, and balance beam test, the motor performance of the mice injected with α‐syn PFFs was worse than that of the control mice, and this effect was attenuated by PLX3397 treatment (Figure S3E–H, Supporting Information). These results suggest that microglia participate in the propagation of α‐syn pathology.

To further verify the effect of microglial depletion on the progression of α‐syn pathology, we used CX_3_CR1cre^ERT2^ × iDTR mice, whose microglia express the diphtheria toxin receptor (DTR) when tamoxifen is administered. One month after the administration of tamoxifen, the mice were treated with diphtheria toxin (DTx) to specifically deplete microglia. In agreement with the previous report,^[^ ^30^ ^]^ the mice presented a marked reduction in microglia in the brain at 1 day after DTx treatment (Figure S4A, Supporting Information). Then, α‐syn PFFs were injected into the striatum. The mice were treated with tamoxifen and DTx every two weeks. α‐Syn pathology was detected at 3 mpi (Figure S4B, Supporting Information). Consistent with what we observed in PLX3397‐treated mice, depletion of microglia in CX_3_CR1cre^ERT2^ × iDTR mice also alleviated α‐syn pathology (Figure S4C–F, Supporting Information). Taken together, these results suggest that specific depletion of microglia partially attenuates α‐syn pathology.

### 2.2 Microglia Process α‐Syn PFFs and Enhance their Seeding Activity

**Figure 2:**
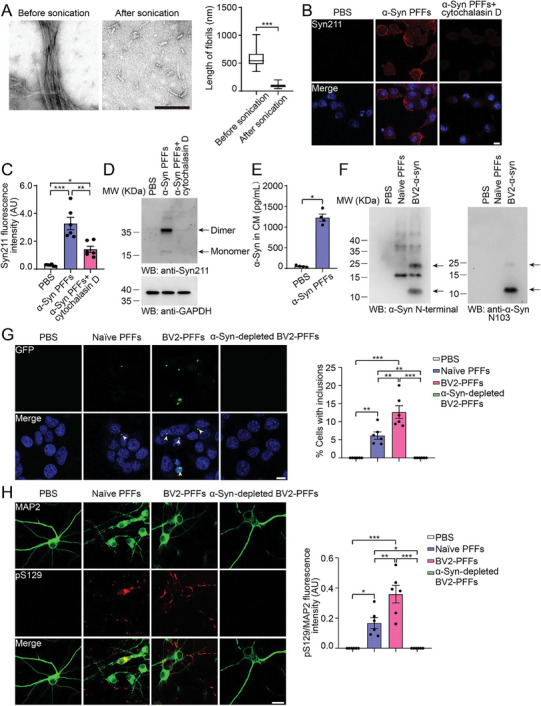
Microglia process α‐syn fibrils and promote their spreading. A) The ultrastructure of α‐syn PFFs before and after sonication (left panel). Scale bar, 200 nm. Right, the lengths of the PFFs (mean ± s.e.m.; n = 50 fibrils in 4 independent experiments; ***p < 0.001; Mann‐Whitney test). B) Immunofluorescence showing the uptake of α‐syn PFFs by BV2 cells in the presence or absence of cytochalasin D. Syn211, red; DAPI, blue. Scale bar, 10 µm. C) Quantification of α‐syn uptake by BV2 cells in the presence or absence of cytochalasin D (mean ± s.e.m.; n = 6 independent experiments; *p < 0.05; **p < 0.01; ***p < 0.001; one‐way ANOVA). D) Western blots showing the levels of endocytosed His‐tagged α‐syn using syn211 antibody. E) ELISA quantification of α‐syn in the conditioned medium of BV2 cells treated with PBS or α‐syn PFFs (mean ± s.e.m.; n = 4 independent experiments; *p < 0.05, Mann‐Whitney test). F) Western blots using α‐syn N‐terminal antibody and N103 antibody showing the bands of α‐syn species in the conditioned medium from α‐syn PFF‐treated BV2 cells. G) Insoluble α‐syn aggregates in α‐syn‐HEK293 cells transduced with naïve α‐syn PFFs or conditioned medium from α‐syn PFF‐treated BV2 cells. The conditioned medium was depleted with or without α‐syn antibody. The cells were treated with 1% TX‐100 to eliminate the soluble α‐syn (mean ± s.e.m.; n = 6 independent experiments; **p < 0.01; one‐way ANOVA). α‐Syn aggregates, green; DAPI, blue. Scale bar, 10 µm. H) Phosphorylation of α‐syn in neurons transduced with naïve α‐syn PFFs and conditioned medium from PFF‐treated BV2 cells. The conditioned medium was depleted with or without α‐syn antibody. The amount of α‐syn species was normalized using ELISA. pS129 signal intensity was normalized to MAP2 (mean ± s.e.m.; n = 6 independent experiments. *p < 0.05; **p < 0.01; ***p < 0.001; one‐way ANOVA). MAP2, green; pS129, red. Scale bar, 20 µm. AU, arbitrary unit. See also in Figures S5 and S6 (Supporting Information).

To investigate the role of microglia in the spread of pathological α‐syn in vitro, we added sonicated α‐syn PFFs (**Figure** **2**A) to the culture medium of BV2 cells, and found that they were taken up by the cells. The phagocytosis of α‐syn PFFs was abolished by the phagocytosis inhibitor cytochalasin D (Figure 2B–D). Interestingly, α‐syn was detected in the conditioned medium of α‐syn PFF‐treated BV2 cells (Figure 2E), indicating that α‐syn PFFs undergo endocytosis and secretion by microglia. Furthermore, Western blot analysis revealed some fragmented α‐syn bands in the conditioned medium, indicating that the α‐syn fibrils are proteolytically cleaved in BV2 cells (Figure 2F). Notably, the BV2‐processed α‐syn fibrils were more resistant to proteinase K (PK) digestion than naïve fibrils (Figure S5A, Supporting Information).

To compare the seeding activity of BV2‐processed α‐syn fibrils and naïve fibrils, we transduced them into HEK293 cells stably transfected with GFP‐α‐syn (α‐syn‐HEK293 cells). As reported previously,^[^ ^31^ ^]^ transduction of α‐syn PFFs induced the formation of Triton X‐100‐insoluble aggregates, which were immunoreactive for pS129 and p62. However, equal amounts of α‐syn species from the conditioned medium of BV2 cells induced more inclusions in the reporter cells than the naïve α‐syn PFFs (Figure 2G; Figure S5B–E, Supporting Information). Sequential extraction of proteins revealed that BV2‐processed α‐syn PFFs induced more Triton X‐100‐insoluble α‐syn species than naïve PFFs (Figure S5F, Supporting Information). Moreover, BV2‐processed α‐syn fibrils also induced more α‐syn phosphorylation than naïve α‐syn fibrils in primary neurons (Figure 2H). Deletion of α‐syn in the conditioned medium blocked the seeding of α‐syn (Figure 2G,H). To confirm the role of microglia in α‐syn pathology, we repeated the experiments with primary microglia and found that α‐syn PFFs were phagocytosed by primary microglia, which was inhibited by cytochalasin D (Figure S6A, Supporting Information). As expected, the α‐syn species processed by primary microglia showed greater seeding activity in both α‐syn‐HEK293 cells and primary neurons compared with naïve α‐syn PFFs (Figure S6B,C, Supporting Information). These results suggest that microglia phagocytose α‐syn PFFs and secrete modified α‐syn species with enhanced seeding activity.

### 2.3 TREM2 Mediates the Phagocytosis of α‐Syn PFFs by Microglia

**Figure 3:**
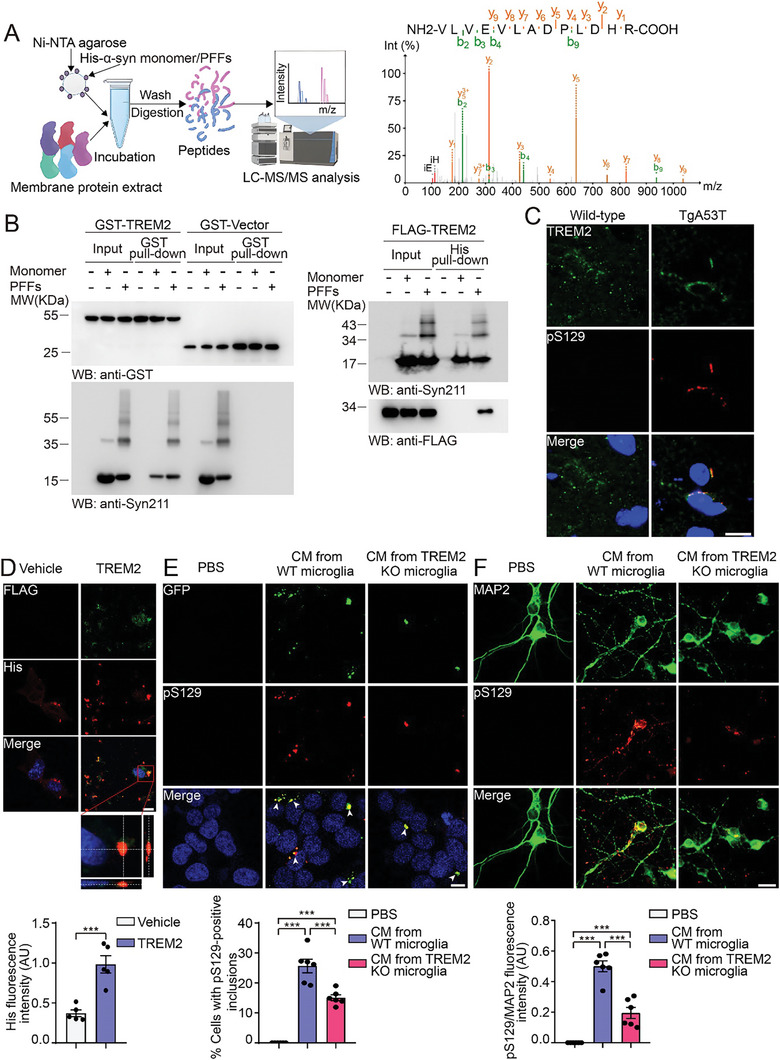
TREM2 mediates the phagocytosis of α‐syn PFFs by microglia. A) Mass spectrometry analysis of the membrane proteins that interact with α‐syn PFFs. The schematic representation of the AP‐MS workflow is shown (left panel). TREM2 was identified by MS (right panel). LC‐MS/MS, liquid chromatography‐tandem mass spectrometry. B) GST pull‐down and His pull‐down assays showing the interaction between α‐syn PFFs and TREM2. His‐tagged α‐syn monomers and PFFs were incubated with cell lysates containing GST‐TREM2 (left panel) or FLAG‐TREM2 (right panel). C) Immunofluorescence showing the colocalization of TREM2 and pS129 in substantia nigra sections from wild‐type and TgA53T mice. TREM2, green; pS129, red; DAPI, blue. Scale bar, 10 µm. D) Phagocytosis of α‐Syn PFFs by BV2 cells overexpressing FLAG‐tagged TREM2 (mean ± s.e.m.; n = 5 independent experiments, ***p < 0.001; *t*‐test). FLAG, green; His, red. Scale bar, 10 µm. E) α‐Syn‐HEK293 cells were incubated with conditioned medium from wild‐type microglia or TREM2 KO microglia that were pretreated with α‐syn PFFs (mean ± s.e.m.; n = 6 independent experiments; ***p < 0.001; one‐way ANOVA). Shown are the insoluble α‐syn aggregates (green). pS129, red; DAPI, blue. Scale bar, 10 µm. F) Phosphorylation of α‐syn in primary neurons induced by conditioned medium (mean ± s.e.m.; n = 6 independent experiments; ***p < 0.001; one‐way ANOVA). Scale bar, 20 µm. AU, arbitrary unit. See also in Figure S7 (Supporting Information).

To further explore the potential membrane proteins that mediate the phagocytosis of α‐syn PFFs by microglia, we performed affinity purification experiments using recombinant His‐tagged α‐syn. His‐α‐syn monomers and PFFs were incubated with membrane proteins prepared from microglia, followed by mass spectrometric analysis of the bound proteins. TREM2 was shown to bind to α‐syn PFFs (**Figure** **3**A; Figure S7A and Table S1, Supporting Information). GST pull‐down and His pull‐down assays confirmed that TREM2 preferentially binds α‐syn PFFs (Figure 3B). Immunofluorescence staining revealed that TREM2 colocalized with pS129 in the substantia nigra of TgA53T mice (Figure 3C; Figure S7B, Supporting Information). To investigate whether TREM2 facilitates the uptake of α‐syn PFFs, we added α‐syn PFFs to BV2 cells overexpressing FLAG‐tagged TREM2. As expected, more α‐syn was observed in cells overexpressing TREM2 (Figure 3D). We further tested the uptake of α‐syn PFFs by primary microglia derived from wild‐type mice and TREM2 KO mice. Consistently, the uptake of α‐syn PFFs was decreased in TREM2 KO microglia compared with that in wild‐type microglia, suggesting that TREM2 facilitates the endocytosis of α‐syn PFFs (Figure S7C, Supporting Information). Furthermore, conditioned medium from wild‐type microglia was more potent than that from TREM2 KO microglia in inducing α‐syn aggregation when transferred into α‐syn‐HEK293 cells (Figure 3E). Similar results were observed when the conditioned medium was transferred into primary neurons (Figure 3F).

### 2.4 Deletion of TREM2 Attenuates the Spread of Pathological α‐Syn In Vivo

We further investigated the role of TREM2 in the spread of α‐syn pathology in TREK2 KO mice. First, we tested whether TREM2 depletion affects the cellular composition of the brain and the cellular state of microglia and astrocytes. We analyzed single‐cell RNA‐seq data from the brains of wild‐type and TREM2 KO mice in a previous report,^[^ ^32^ ^]^ and found that the cellular composition of the brain was not altered in TREM2 KO mice (Figure S8A–C, Supporting Information). Furthermore, the composition of microglial clusters was not altered in TREM2 KO mice (Figure S8D‒F, Supporting Information). Similarly, the composition of the astrocyte clusters was not altered (Figure S8G–I, Supporting Information).

**Figure 4:**
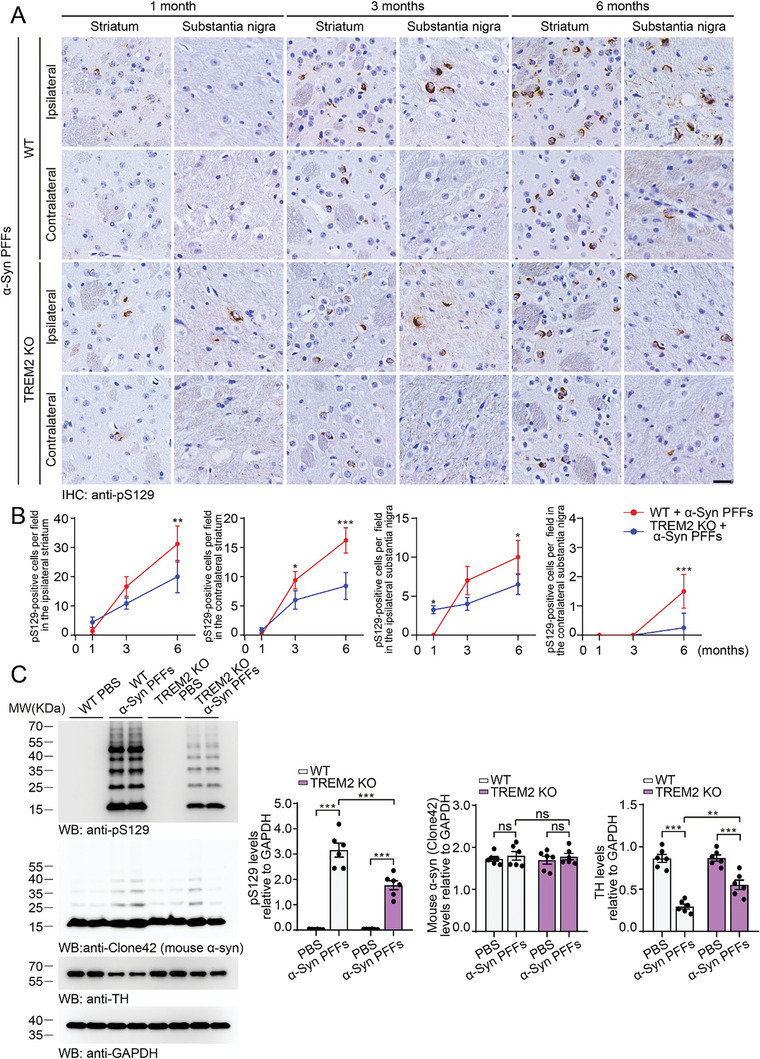
Deletion of TREM2 attenuates the spreading of α‐syn in vivo. A) Representative images of α‐syn pathology in wild‐type and TREM2 KO mice at 1, 3, and 6 months after injection of α‐syn PFFs. Scale bar, 20 µm. B) The quantification of pS129‐positive signals in the striatum and substantia nigra (mean ± SD; n = 4–5 mice per group, 6 visual fields in each mouse; *p < 0.05; **p < 0.01; ***p < 0.001; two‐way ANOVA). C) Western blots showing the levels of pS129, mouse α‐syn (Clone 42 antibody), and TH in the striatum of wild‐type and TREM2 KO mice injected with PBS or α‐syn PFFs at 6 mpi (mean ± s.e.m.; n = 6 mice per group; ***p < 0.001; two‐way ANOVA). ns, not significant. See also in Figures S8–S12 (Supporting Information).

Equal amounts of α‐syn PFFs were injected into the right striatum of wild‐type mice and TREM2 KO mice. Three days after injection, exogenous α‐syn PFFs were found mainly in microglia from wild‐type mice. Knockout of TREM2 dramatically decreased the levels of α‐syn PFFs in microglia, indicating that the microglial phagocytosis of α‐syn PFFs was attenuated by the deletion of TREM2 (Figure S9, Supporting Information). α‐Syn pathology was examined at 1, 3, and 6 mpi. At 1 mpi, the density of pS129 signals in the substantia nigra ipsilateral to the injection site was slightly higher in TREM2 KO mice than in wild‐type mice. pS129 signals were detected in the ipsilateral striatum and substantia nigra in both mouse lines at 3 mpi and progressed to the contralateral hemisphere at 6 mpi. The extent of α‐syn pathology was much lower in TREM2 KO mice than in wild‐type mice at 6 mpi (**Figure** **4**A,B). Consistently, more high‐molecular‐weight α‐syn species were detected in the ipsilateral striatum of PFF‐injected wild‐type mice than in PFF‐injected TREM2‐KO mice. Furthermore, the expression levels of TH in the striatum were greater in TREM2 KO mice than in wild‐type mice (Figure 4C).

The density of microglia in the ipsilateral striatum and substantia nigra increased in a time‐dependent manner and was attenuated in TREM2 KO mice (Figure S10A, B, Supporting Information). We also tested the levels of pro‐inflammation factors in wild‐type and TREM2 KO mice after the injection of α‐syn PFFs. The levels of IL‐1β, IL‐6 and TNF‐α were elevated after α‐syn PFF injection. The degree of elevation was greater in the wild‐type mice than in the TREM2 KO mice (Figure S10C, Supporting Information). Moreover, more pS129 α‐syn signals were detected in the microglia of PFF‐injected wild‐type mice than in those of TREM2 KO mice (Figure S11A, B, Supporting Information). Similarly, the brain lysates from TREM2 KO mice showed decreased seeding activity compared with those from wild‐type mice when added to α‐syn monomers (Figure S11C, Supporting Information).

**Figure 5:**
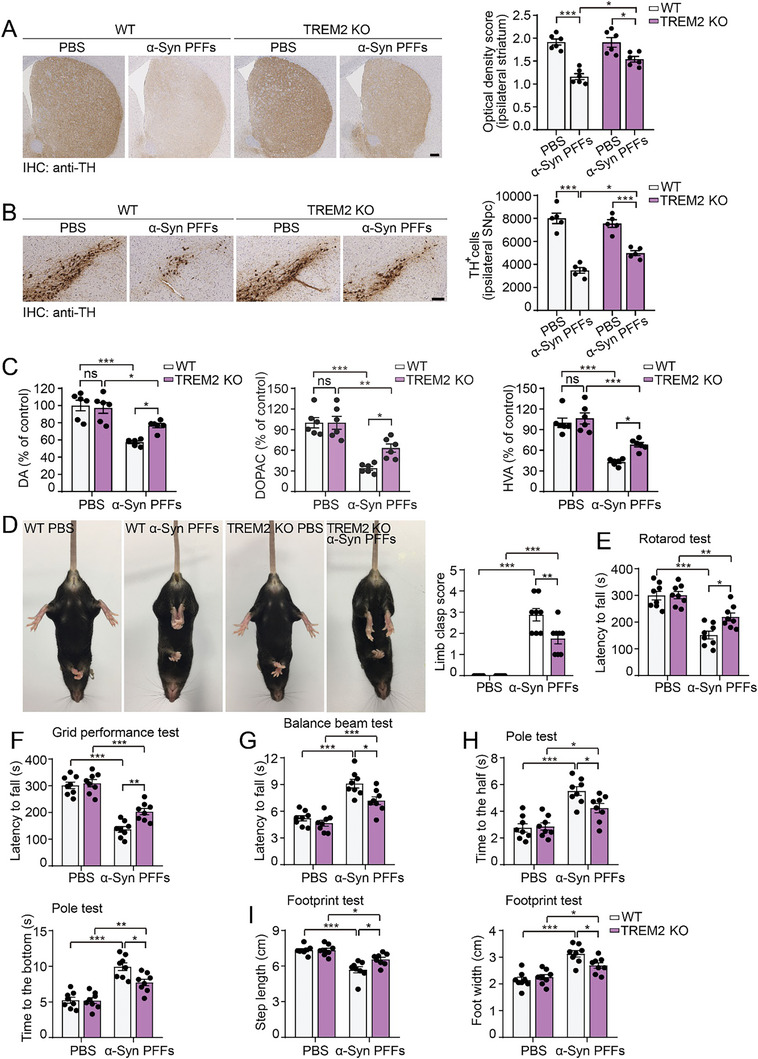
Deletion of TREM2 attenuates dopaminergic neurodegeneration and motor impairment. A) TH immunostaining of the ipsilateral striatum and quantification of the average optical density of wild‐type and TREM2 KO mice injected with PBS or α‐syn PFFs at 6 mpi (mean ± s.e.m.; n = 6 mice per group; *p < 0.05; ***p < 0.001; two‐way ANOVA). Scale bar, 200 µm. B) Representative images of dopaminergic neurons in the ipsilateral SNpc region of wild‐type and TREM2 KO mice injected with PBS or α‐syn PFFs at 6 mpi (mean ± s.e.m.; n = 5 mice per group; *p < 0.05; ***p < 0.001; two‐way ANOVA). Scale bar, 100 µm. C) HPLC analysis of striatal DA, DOPAC, and HVA in mice injected with PBS or α‐syn PFFs at 6 mpi (mean ± s.e.m.; n = 6 mice per group; *p < 0.05; **p < 0.01; ***p < 0.001; two‐way ANOVA). ns, not significant. D) Representative images of the tail suspension test at 6 mpi in each group (mean ± s.e.m.; n = 8 mice per group; **p < 0.01; ***p < 0.001; two‐way ANOVA). E–I) The rotarod test, grid performance test, balance beam test, pole test, and footprint test were performed at 6 mpi in wild‐type and TREM2 KO mice (mean ± s.e.m.; n = 8 mice per group; *p < 0.05; **p < 0.01; ***p < 0.001; two‐way ANOVA).

We further compared degeneration of the nigrostriatal dopaminergic pathway in wild‐type and TREM2 KO mice injected with α‐syn PFFs. More severe loss of dopaminergic terminals in the ipsilateral striatum was observed in wild‐type mice than in TREM2 KO mice at 6 mpi (**Figure** **5**A). Similarly, we found a more severe loss of dopaminergic neurons in the ipsilateral substantia nigra in wild‐type mice (Figure 5B). The levels of dopamine and its metabolites were decreased in α‐syn PFF‐injected wild‐type mice but attenuated in TREM2 KO mice (Figure 5C). Furthermore, compared with wild‐type mice, TREM2 KO mice presented improved motor performance in the tail suspension test, rotarod test, grid performance test, balance beam test, pole test, and footprint test (Figure 5D–I). Therefore, TREM2 KO partially ameliorates the degeneration of dopaminergic neurons and PD‐like motor impairments induced by α‐syn PFFs.

To further confirm the role of TREM2 in α‐syn pathology, we reconstituted the expression of TREM2 in TREM2 KO mice. The mice were intrastriatally injected with AAVs encoding TREM2 or control AAVs, together with α‐syn PFFs. As expected, α‐syn pathology was more pronounced in the mice injected with AAV‐TREM2 than in the mice injected with control AAVs (Figure S12A–C, Supporting Information). These results further support that TREM2 facilitates the progression of α‐syn pathology in the brain.

### 2.5 AEP Cleaves α‐Syn Fibrils and Generates more Toxic Fibrils

**Figure 6:**
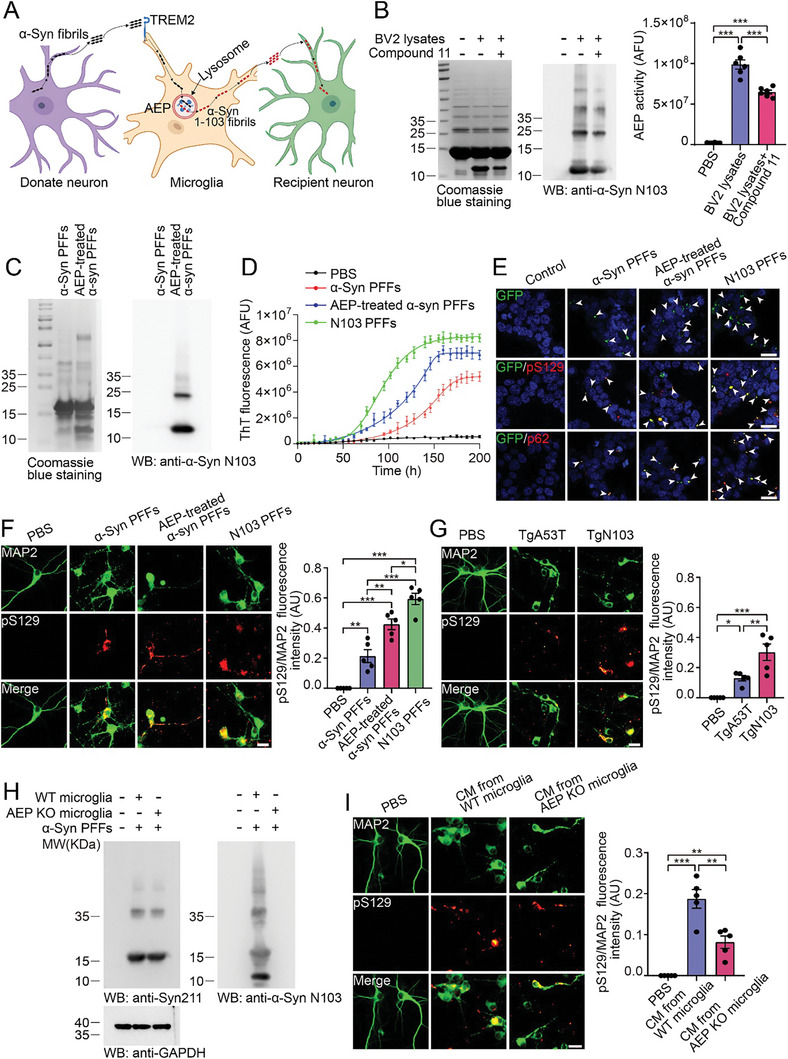
AEP cleaves α‐syn PFFs in microglia and generates a more toxic strain. A) The hypothesis diagram showing that microglia mediate the spread of pathological α‐syn. B) Coomassie blue staining (left) and Western blots (right) of α‐syn PFFs incubated with BV2 cell lysates. The bar graph shows AEP activity (mean ± s.e.m.; n = 6 independent experiments; ***p < 0.001; one‐way ANOVA). C) Coomassie blue staining and Western blot analysis of α‐syn PFFs treated with AEP. D) The aggregation kinetics of α‐syn (0.75 mg mL^−1^) seeded with α‐syn PFFs, AEP‐treated α‐syn PFFs, and recombinant N103 PFFs. The starting seeds were normalized by ELISA quantification of the α‐syn concentration. Nonlinear regression was used to generate sigmoid‐shaped curves. AFU: arbitrary fluorescence unit. E) Insoluble α‐syn aggregates in α‐syn‐HEK293 cells induced by α‐syn PFFs, AEP‐treated α‐syn PFFs, and N103 PFFs. The cells were treated with 1% Triton X‐100 to eliminate soluble α‐syn. The aggregates colocalize with pS129 and p62. α‐Syn aggregates, green; pS129/p62, red; DAPI, blue. Scale bar, 25 µm. F) Phosphorylation of α‐syn induced by α‐syn PFFs, AEP‐treated α‐syn PFFs, and N103 PFFs in primary neurons (mean ± s.e.m.; n = 5 independent experiments; *p < 0.05; **p < 0.01; ***p < 0.001; one‐way ANOVA). MAP2, green; pS129, red. Scale bar, 20 µm. G) The phosphorylation of α‐syn in primary neurons seeded with brain extracts from 12‐month‐old TgA53T mice or TgN103 mice (mean ± s.e.m.; n = 5 independent experiments; *p < 0.05; **p < 0.01; ***p < 0.001; one‐way ANOVA). MAP2, green; pS129, red. Scale bar, 20 µm. H) Western blots showing the levels of α‐syn and the N103 fragment in α‐syn PFF‐treated wild‐type and AEP KO microglia. I) Phosphorylation of α‐syn induced by conditioned medium from α‐syn PFF‐treated wild‐type and AEP KO microglia (mean ± s.e.m.; n = 5 independent experiments; **p < 0.01; ***p < 0.001; one‐way ANOVA). MAP2, green; pS129, red. Scale bar, 20 µm. See also in Figures S13 and S14 (Supporting Information).

Based on the above results, we hypothesized that microglia endocytose α‐syn fibrils and process them into more toxic fibrils, facilitating the spread of pathological α‐syn (**Figure** **6**A). To test this hypothesis, we incubated His‐tagged α‐syn PFFs with BV2 cell lysates and purified the α‐syn species using Ni‐NTA agarose. Coomassie blue staining revealed a novel fragment of α‐syn with a molecular weight of ≈12 kDa (Figure 6B). We previously reported that α‐syn is cleaved by the lysosomal protease asparagine endopeptidase (AEP), generating the α‐syn N103 fragment, which forms fibrils with enhanced seeding activity and neurotoxicity compared with those of full‐length α‐syn.^[^ ^11^ ^]^ Western blot analysis using an antibody specific for the N103 fragment revealed that incubating α‐syn PFFs with BV2 cell lysates induced the appearance of the N103 fragment, which was partially blocked by the AEP inhibitor compound 11^[^ ^33^ ^]^ (Figure 6B). Furthermore, the α‐syn N103 fragment was detected when α‐syn PFFs were incubated with wild‐type microglial lysates but not with AEP KO microglial lysates (Figure S13, Supporting Information). The N103 fragment was also detected in the conditioned medium of α‐syn PFF‐treated BV2 cells (Figure 2F). To further verify whether AEP directly processes α‐syn PFFs, we incubated α‐syn PFFs with recombinant AEP. As expected, incubation with AEP induced the generation of the N103 fragment (Figure 6C). Moreover, the N103 fragment was detected in BV2 cells exposed to α‐syn fibrils but was diminished in the presence of the phagocytosis inhibitor cytochalasin D (Figure S14A, B, Supporting Information). The α‐syn N103 fragment was also detected in the cell lysates of primary microglia treated with α‐syn PFFs but was strongly decreased in microglia cultured from TREM2 KO mice (Figure S14C, Supporting Information). Collectively, AEP in microglia cleaves α‐syn PFFs and generates α‐syn N103 fibrils. Interestingly, PK digestion analysis revealed that α‐syn PFFs formed by the α‐syn N103 fragment were more resistant to protease digestion than PFFs formed by full‐length α‐syn (Figure S14D, Supporting Information).

To test the effect of AEP‐mediated cleavage on the seeding activity of α‐syn PFFs, we added equal amounts of α‐syn PFFs, AEP‐treated α‐syn PFFs, and α‐syn PFFs generated from the recombinant N103 fragment (N103 PFFs) to α‐syn monomers and monitored the aggregation kinetics via the thioflavin T (ThT) assay. We found that AEP‐treated α‐syn PFFs and N103 PFFs were more potent at promoting the aggregation of α‐syn monomers than were full‐length α‐syn PFFs (Figure 6D). When these fibrils were transduced into α‐syn‐HEK293 cells, more Triton X‐100 (TX‐100)‐insoluble aggregates were observed in cells transduced with AEP‐processed α‐syn PFFs and N103 PFFs than in cells treated with full‐length α‐syn PFFs (Figure 6E). Similar results were found when PFFs were transduced into primary neurons (Figure 6F). Compared with α‐syn PFFs, α‐Syn N103 PFFs presented stronger neurotoxicity (Figure S14E, Supporting Information). Furthermore, we compared the seeding activity of brain extracts from 12‐month‐old TgA53T transgenic mice that express human A53T mutant α‐syn and TgN103 mice that express the human α‐syn N103 fragment.^[^ ^34^ ^]^ More pS129 signals were detected in neurons exposed to TgN103 brain extracts than in neurons exposed to TgA53T brain extracts (Figure 6G). These results collectively support that AEP‐generated N103 PFFs are more potent at promoting α‐syn aggregation.

To further verify the role of AEP in the generation of the N103 fragment, primary microglia from wild‐type and AEP KO mice were incubated with α‐syn PFFs. The uptake of α‐syn PFFs was similar between the wild‐type and AEP KO microglia, while α‐syn N103 was detected only in the conditioned medium from the wild‐type microglia and not in that from the AEP KO microglia (Figure 6H). When conditioned medium was added to primary neurons, the wild‐type medium induced more pS129 signals than the AEP KO medium did (Figure 6I). Thus, microglial AEP processes α‐syn aggregates, generating α‐syn N103 fibrils with increased seeding activity.

### 2.6 Deletion of AEP Attenuates the Propagation of α‐Syn In Vivo

**Figure 7:**
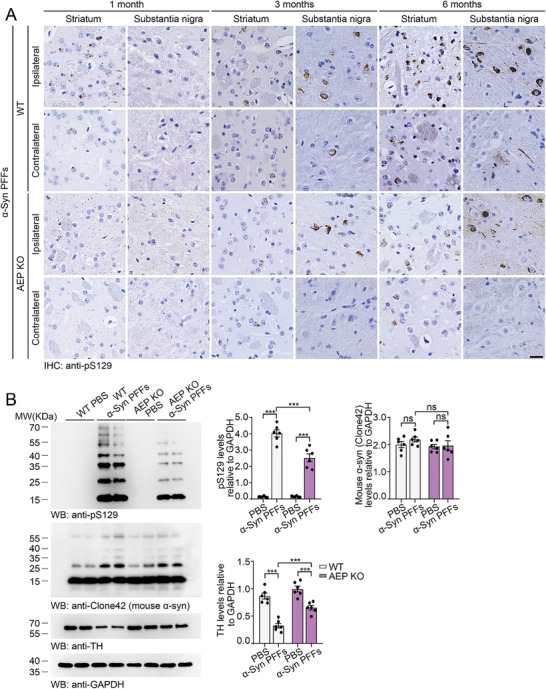
Deletion of AEP attenuates the aggregation of α‐syn in vivo. A) Representative images of α‐syn pathology in wild‐type and AEP KO mice injected with α‐syn PFFs at 1, 3, and 6 mpi. Scale bar, 20 µm. B) Western blot analysis showing the levels of pS129, mouse α‐syn (clone 42 antibody), and TH in the striatum of wild‐type and AEP KO mice injected with PBS or α‐syn PFFs at 6 mpi (mean ± s.e.m.; n = 6 mice per group; ***p < 0.001; two‐way ANOVA). ns, not significant. See also in Figures S15–S17 (Supporting Information).

**Figure 8:**
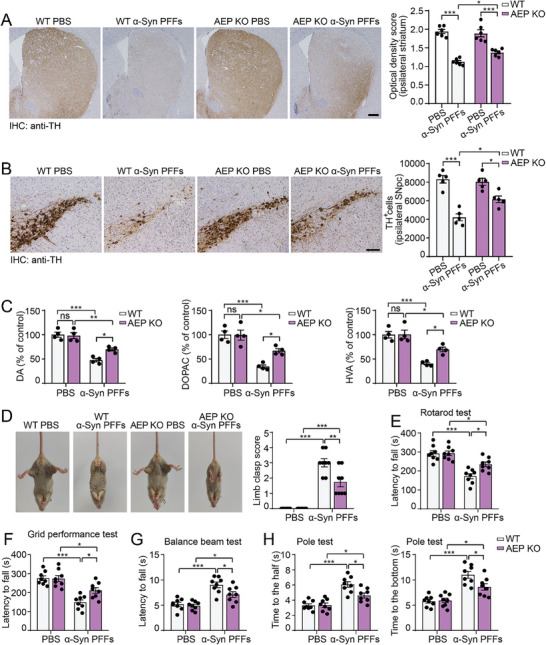
Deletion of AEP ameliorates dopaminergic neurodegeneration. A) TH immunostaining of the ipsilateral striatum and quantification of the average optical density at 6 mpi (mean ± s.e.m.; n = 6 mice per group; *p < 0.05; ***p < 0.001; two‐way ANOVA). Scale bar, 100 µm. B) Representative images of dopaminergic neurons in the ipsilateral SNpc of wild‐type and AEP KO mice injected with PBS or α‐syn PFFs at 6 mpi (mean ± s.e.m.; n = 5 mice per group; *p < 0.05; ***p < 0.001; two‐way ANOVA). Scale bar, 100 µm. C) HPLC analysis of striatal DA, DOPAC, and HVA in mice treated with PBS or α‐syn PFFs at 6 mpi (mean ± s.e.m.; n = 4 mice per group; *p < 0.05; **p < 0.01; ***p < 0.001; two‐way ANOVA). ns, not significant. D) Representative images of the tail suspension test at 6 mpi in each group (mean ± s.e.m.; n = 8 mice per group; **p < 0.01; ***p < 0.001; two‐way ANOVA). E‐H) The rotarod test, grid performance test, balance beam test, and pole test were performed at 6 mpi in wild‐type and AEP KO mice (mean ± s.e.m.; n = 8 mice per group; *p < 0.05; ***p < 0.001; two‐way ANOVA).

To investigate whether AEP regulates the progression of α‐syn pathology in vivo, we injected α‐syn PFFs into the right striatum of wild‐type mice and AEP KO mice. The density of α‐syn inclusions positive for pS129 and ubiquitin was much lower in the AEP KO mice than in the wild‐type mice at 1, 3, and 6 mpi (**Figure** **7**A; Figures S15 and S16A, Supporting Information). pS129 signals were detected in microglia from both PFF‐injected wild‐type and AEP KO mice (Figure S16B, Supporting Information). The microglial density in the ipsilateral striatum of PFF‐injected wild‐type mice was higher than that in AEP KO mice (Figure S17A, B, Supporting Information). We also tested the levels of pro‐inflammation factors in wild‐type and AEP KO mice after the injection of α‐syn PFFs. The levels of IL‐1β, IL‐6, and TNF‐α were elevated after α‐syn PFF injection, whereas AEP knockout alleviated the increase in these pro‐inflammatory factors (Figure S17C, Supporting Information). Consistently, high‐molecular‐weight α‐syn species in the ipsilateral striatum were more abundant in wild‐type mice than in AEP KO mice. Knockout of AEP partially ameliorated the loss of striatal TH induced by α‐syn PFFs (Figure 7B). Moreover, the loss of dopaminergic terminals in the striatum and dopaminergic neurons in the SN was also partially attenuated in AEP KO mice at 6 mpi (**Figure** **8**A,B). The decrease in dopamine and dopamine metabolites in the ipsilateral striatum was partially reversed in AEP KO mice (Figure 8C). As expected, behavioral tests revealed that the motor impairment induced by α‐syn PFFs was partially ameliorated by AEP knockout (Figure 8D–H). These results suggest that deletion of AEP attenuates the propagation of α‐syn pathology, dopaminergic neuronal degeneration, and PD‐like motor impairment.

## 3 Discussion

In this study, we revealed that the microglial receptor TREM2 interacts with α‐syn fibrils and mediates their phagocytosis. The lysosomal protease AEP subsequently cleaves α‐syn fibrils and generates N103 fibrils, which exhibit enhanced seeding activity. Knockout of TREM2 inhibits the endocytosis of α‐syn PFFs by microglia, alleviating the α‐syn pathology and motor dysfunction induced by the injection of exogenous α‐syn PFFs. Knockout of AEP does not affect the phagocytosis process but inhibits the production of N103 species and the spread of pathological α‐syn. Thus, TREM2 mediates the endocytosis of pathological α‐syn by microglia, whereas AEP mediates the proteolytic processing of α‐syn PFFs in microglia. These two steps contribute to the propagation of α‐syn pathology.

The propagation and transmission of pathological α‐syn underlie the stereotypical progression of pathology through the brain over time in PD patients. Converging evidence suggests that microglia participate in the onset and progression of PD pathology. However, the exact molecular mechanisms remain elusive.^[^ ^35^, ^36^ ^]^ Pathological α‐syn has been reported to interact with microglia and trigger pathological responses in microglia, which can be attributed to the hydrolysis resistance of α‐syn fibrils.^[^ ^17^ ^]^ Our results indicate that microglia phagocytose α‐syn fibrils and process them into α‐syn species with increased seeding activity. These data are supported by previous observations that microglia are involved in the spread of pathological α‐syn.^[^ ^37^, ^38^ ^]^

Here we identified TREM2 as a receptor that mediates the phagocytosis of α‐syn PFFs by microglia. Deletion of TREM2 blocks the uptake of α‐syn PFFs. Interestingly, one study reported that deletion of TREM2 aggravated neurodegeneration in mice injected with AAV‐α‐syn at 3–8 mpi.^[^ ^39^ ^]^ Another study revealed that TREM2 overexpression attenuates neuroinflammation and protects dopaminergic neurons in an MPTP‐induced mouse model of PD.^[^ ^40^ ^]^ These results suggest that TREM2 may exert different effects under different conditions. Furthermore, TREM2‐mediated endocytosis of α‐syn PFFs by microglia plays distinct roles in different phases of disease progression. In the early stage, TREM2 mediates the phagocytosis of pathological α‐syn species by microglia and may promote their clearance. However, at the late stage, the endocytosed α‐syn species may overwhelm the degradation capacity of microglia and aggregate in the endolysosomal system, where they are partially processed by AEP to generate α‐syn N103 species with enhanced seeding activity.

AEP is a lysosomal cysteine protease that specifically cleaves its substrates at the C‐terminal side of asparagine (N) residues.^[^ ^41^, ^42^ ^]^ AEP is upregulated and activated during aging and in the brains of patients with PD. It cleaves α‐syn at the N103 residue in an age‐dependent manner and generates N103 fragments.^[^ ^11^ ^]^ α‐Syn fibrils formed by the N103 fragment are more toxic than those formed by full‐length α‐syn.^[^ ^11^ ^]^ We generated a transgenic mouse line expressing the α‐syn N103 fragment and reported that the N103 fragment induces the loss of dopaminergic neurons in the substantia nigra and motor impairments.^[^ ^34^ ^]^ Here, we showed that not only α‐syn monomers but also α‐syn fibrils can be cleaved by AEP in microglia, generating N103 fibrils. Cryo‐electron microscopy (cryo‐EM) revealed that the α‐syn fibrils contain residues 37–99, while approximately 40 residues at both the N‐ and C‐terminal ends of full‐length α‐syn surround the structured core of the fibril, forming the “fuzzy coat”.^[^ ^43^, ^44^ ^]^ Cleavage of α‐syn PFFs by AEP removes the C‐terminal “fuzzy coat” and generates distinct fibrils. Emerging evidence supports the presence of conformationally diverse strains of α‐syn in the brain, contributing to the pathological and clinical heterogeneity of synucleinopathies.^[^ ^45^, ^46^, ^47^, ^48^ ^]^ AEP‐mediated cleavage of α‐syn fibrils may represent one of the mechanisms by which microglia generate different α‐syn strains in the brain. We observed strong protective effects of AEP knockout and TRME2 knockout in the PFFs‐induced mouse model of PD, as shown by consistent results in various behavioral studies. In conclusion, we identified TREM2 and AEP as key regulators that promote the progression of α‐syn pathology. Targeting TREM2 and AEP could point to a new therapeutic approach for synucleinopathies, including PD.

*Limitations of this Study*: Some limitations of the study need to be acknowledged. First, we used whole‐body AEP KO mice to demonstrate the role of AEP in α‐syn pathology. Notably, in addition to microglia, AEP is also expressed in neurons, astrocytes, and oligodendrocytes, which may also contribute to the progression of α‐syn pathology. Second, we injected synthetic α‐syn PFFs into the mouse brain to induce the spread of pathological α‐syn. It has been reported that synthetic PFFs may cause pathology distinct from that of brain‐derived α‐syn fibrils.^[^ ^49^ ^]^ Third, the deletion of microglia, TREM2, or AEP only partially attenuated motor impairments, indicating that other pathways also contribute to α‐syn pathology.

## 4 Experimental Section

### Mice

The TREM2 KO mice were from Cyagen (No. KOCMP‐83433‐Trem2‐B6N‐VA). The AEP KO mice were gifts from Dr. Hara‐Nishimura from the National Institute for Basic Biology, Japan.^[^ ^50^ ^]^ CX_3_CR1cre^ERT2^ × iDTR mice were generated by crossing CX_3_CR1‐CreERT2 mice (C001247, Cyagen Bioscience) with ROSA26‐LSL‐DTR mice (C001189, Cyagen Biosciences). Mice were housed under specific pathogen‐free conditions on a 14 h light/10 h dark cycle. Experimental procedures were approved by the Laboratory Animal Welfare and Ethics Committee of Renmin Hospital of Wuhan University, with ethical approval number 20200525. All animal care and handling were performed according to the Declaration of Helsinki. The mice were randomized into different groups. Investigators were blinded to the group allocation during the animal experiments.

### Antibodies and Reagents

The following antibodies were used in this study: Iba1 (Wako, 019–19741), MAP2 (Proteintech, 17490‐1‐AP), GFP (Proteintech, 66002‐1‐Ig), GFAP (Invitrogen, PA5‐16291), P2Y12 (Absin, abs121674a), GAPDH (Proteintech, 60004‐1‐Ig), phospho‐α‐syn (Ser129) (BioLegend, 825 701), phospho‐α‐syn (Ser129) (Cell Signaling Technology, 23 706), α‐Syn (Clone 42, BD Biosciences, 610 786), α‐syn (Syn211, Invitrogen, MA5‐12272), α‐syn (N‐terminal, from Gene Science Pharmaceuticals Co., Ltd), α‐syn N103 (homemade, reported previously^[^ ^11^ ^]^), p62 (Abcam, 56 416), His (Proteintech,66005‐1‐Ig), FLAG (Proteintech, 20543‐1‐AP), GST (Proteintech, 66001‐2‐Ig), TH (Millipore, AB152), ubiquitin (Cell Signaling Technology, 3936S), HRP‐conjugated anti‐mouse IgG (Bio‐Rad, 170–6516), HRP‐conjugated anti‐rabbit IgG (Bio‐Rad, 170–6515), Alexa Fluor 488‐Goat anti‐Mouse (Invitrogen, A11001), Alexa Fluor 488‐Goat anti‐Rabbit (Invitrogen, A11034), Alexa Fluor 594‐Goat anti‐Mouse (Invitrogen, A11005), Alexa Fluor 594‐Goat anti‐Rabbit (Invitrogen, A11012). The following reagents were used: PLX3397 (MCE, HY‐16749), cytochalasin D (MCE, HY‐N6682), and compound 11 (Santa Cruz Biotechnology, sc‐319780).

### Human Tissue Samples

Postmortem brain sections were obtained from the brain samples of PD patients and control patients at the Emory Alzheimer's Disease Research Center. The study was approved by the biospecimen committee of Emory University (P30NS055077). PD patients were pathologically verified and clinically diagnosed. Informed consent was obtained from all subjects.

### Cell Culture

HEK293 cells, α‐syn‐HEK293 cells, and BV2 cells were cultured in DMEM supplemented with 10% fetal bovine serum and 1% penicillin‐streptomycin at 37 °C in 5% CO_2_/95% air. Primary neuronal cultures were prepared from E18 embryonic mice. The mice were anesthetized and sterilized with 75% ethanol. The brains of the embryos were collected, and the meninges were removed under a dissection microscope. The dissected cortex tissue was collected in a 15‐mL centrifuge tube containing DMEM and 1 × trypsin/EDTA and further triturated by approximately 10 passes through a smooth‐edged normal glass Pasteur pipette. After centrifugation, the cells were plated in 6‐well plates that were pre‐coated with poly‐D‐lysine (PDL) in DMEM containing 6% heat‐activated fetal bovine serum, 6% horse serum, 1% L‐glutamine, and 1% penicillin‐streptomycin. Six hours later, the medium was changed to neurobasal medium supplemented with 2% B‐27, 1% L‐glutamine, and 1% penicillin‐streptomycin.^[^ ^51^ ^]^

To obtain primary microglia, dissected cortical tissue was collected in a 15‐mL centrifuge tube with cold Hank's buffered saline and digested in 1 × trypsin/EDTA solution at 37 °C for 15 min. Complete microglial medium was added to stop the digestion. The cells were then centrifuged at 1000 rpm for 5 min and resuspended in 10 mL of complete microglial medium. The cells were plated in culture dishes and cultured at 37 °C in 5% CO_2_/95% air. The medium was changed on the third day. Seven days after plating, 400 µL of lidocaine (60 mM) was added to the medium, and the mixture was incubated at room temperature for 10 min to detach the cells. The cells were then collected by centrifugation at 1000 r.p.m. for 5 min, resuspended in 1 mL of DMEM supplemented with FBS, and plated into 6‐well plates.

### Purification of Recombinant Human α‐Syn and Preparation of α‐Syn PFFs

Recombinant human α‐syn and the N103 monomers were purified as previously described.^[^ ^52^ ^]^ *Escherichia coli* BL21 cells were transduced with PRK172 plasmids encoding His‐tagged α‐syn, α‐syn (a.a. 1–103), or the N103A mutant α‐syn and cultured in a shaker at 37 °C overnight. The bacteria were harvested by centrifugation at 8000 rpm for 5 min and resuspended in osmotic shock buffer (30 mM Tris‐HCl, 40% sucrose, 2 mM ethylenediaminetetraacetic acid disodium, pH 7.2). The suspension was incubated for 10 min at room temperature and then centrifuged at 12 000 rpm for 20 min. Next, the pellets were resuspended quickly in saturated magnesium chloride solution. After centrifugation at 12 000 rpm for 20 min, the supernatants were collected and subjected to Ni‐chelating affinity chromatography. The column was rinsed with 20 mM imidazole to wash away nonspecific binding and then rinsed with 250 mM imidazole to elute α‐syn. The solution was dialyzed, lyophilized, and then stored at ‐80 °C. To prepare PFFs and perform ThT staining, the lyophilized α‐syn monomers were dissolved in sterile PBS and centrifuged at 100 000 × g for 30 min at 4 °C to remove any preformed insoluble aggregates. The supernatants were collected, mixed with Microcon 30 kDa, and centrifuged at 15 300 × g for 20 min at 4 °C. The filtrate was collected as monomers. The status of the monomers was confirmed using Western blot analysis. The samples were then continuously agitated at 1000 r.p.m. for 5 days at 37 °C in a mixer (Eppendorf, Hamburg, Germany). ThT fluorescence assay was used to detect the kinetics of α‐syn fibrillization. The culture mixture (10 µL) was diluted to 100 µL with 25 mM ThT in PBS and incubated at 37 °C for 5 min in the dark. Fluorescence was detected at an excitation wavelength of 450 nm and an emission wavelength of 510 nm using a Spectra Max plate reader (Molecular Devices, Sunnyvale, CA). α‐Syn PFFs were sonicated with 60 pulses at 10% power (0.5 s on, 0.5 s off) before use. Aliquots of α‐syn PFFs were stored at ‐80 °C before use. The AEP‐treated α‐syn PFFs were prepared by adding active AEP enzyme (Novoprotein, 5 µg mL^−1^) into the α‐syn PFFs and incubated for 30 min at pH 6.0 at 37 °C.

### Stereotaxic Injection of α‐Syn PFFs into the Mouse Brain

The α‐syn PFFs were sonicated and stereotaxically injected into the right striatum (coordinates: anterior‐posterior (AP) + 0.2 mm, medial‐lateral (ML) – 2.0 mm relative to the bregma, dorsoventral (DV) – 2.7 mm from the dural surface) of 2‐month‐old wild‐type, TREM2 KO, and AEP KO mice at a speed of 300 nL min^−1^ (5 µg total protein per brain) by using a 10 µL glass syringe (Hamilton) with a fixed needle. The syringe was held for an additional 5 min to allow complete absorption of the solution. After injection, the mice were monitored and provided with post‐surgery care.

### Administration of Tamoxifen and Diphtheria Toxin

Tamoxifen was dissolved in corn oil at a concentration of 20 mg/mL by shaking overnight at 37 °C. CX_3_CR1cre^ERT2^ × iDTR mice were orally gavaged with tamoxifen (75 mg kg^−1^ body weight) once daily for 3 days. Administration of tamoxifen induces CX_3_CR1‐positive cells to express DTR. Because microglia have a slower turnover rate than monocytes, only microglia continue to express DTR after several weeks.^[^ ^30^ ^]^ Four weeks after the administration of tamoxifen, microglia were selectively depleted by administration of DTx (30 ng g^−1^ body weight, Aladdin) once daily for 3 days prior to the intrastriatal injection of α‐syn PFFs. After injection, tamoxifen and DTx were administered every two weeks.

### Stereotaxic Injection of Virus into the Mouse Brain

AAV particles encoding human A53T mutant α‐syn, mouse TREM2, and mouse AEP were prepared by BrainVTA (BrainVTA Co., Ltd.). AAV‐α‐syn A53T was injected into the right substantia nigra (coordinates: AP −3.1 mm and ML −1.2 mm relative to the bregma, and DV −4.0 mm from the dural surface) of 2‐month‐old wild‐type mice and TREM2 KO mice. AAV‐TREM2 or AAV‐AEP together with α‐syn PFFs were injected into the right striatum (coordinates: AP + 0.2 mm, ML – 2.0 mm relative to bregma, DV – 2.7 mm from the dural surface) of 2‐month‐old TREM2 KO mice or AEP KO mice, respectively. A total of 300 nL of viral suspension was injected into the substantia nigra or striatum with a 10 µL glass syringe with a fixed needle at a rate of 40 nL min^−1^. The needle remained in place for an additional 5 min before it was removed slowly. After injection, the mice were monitored and provided with post‐surgery care.

### Preparation of Sarkosyl‐Insoluble Fractions from Mouse Brain Tissue

Sarkosyl‐insoluble α‐syn was extracted as described previously.^[^ ^53^ ^]^ Briefly, striatal tissues from 12‐month‐old TgA53T mice and TgN103 mice were homogenized in high‐salt (HS) buffer (50 mM Tris‐HCl (pH 7.4), 750 mM NaCl, 10 mM NaF, 5 mM EDTA) supplemented with protease and phosphatase inhibitors, followed by centrifugation at 100 000 × g for 30 min at 4 °C. The pellets were resuspended in 1% Triton X‐100‐containing HS buffer and centrifuged at 100 000 × g for 30 min at 4 °C. The supernatant was discarded. The pellets were re‐extracted in 1% Triton X‐100 HS buffer with 30% sucrose and centrifuged at 100 000 × g for 30 min at 4 °C. The supernatant was discarded, and the pellets were resuspended in 1% sarkosyl‐containing HS buffer, rotated overnight at 4 °C, and then centrifuged at 100 000 × g for 30 min at 4 °C. The pellets were washed in PBS and resuspended in PBS by extensive sonication. This suspension was termed the sarkosyl‐insoluble fraction.

### Transduction of Cells with α‐syn PFFs

α‐syn PFFs were transduced into cells as described previously.^[^ ^54^ ^]^ To transduce α‐syn‐HEK293 cells, α‐syn PFFs (5 µg) were mixed with 3 µL of Lipofectamine 2000 in 250 µL of Opti‐MEM, incubated for 20 min, and subsequently added to one well of six‐well plates. To transduce primary microglia, 5 µg of α‐syn PFFs was added to the medium at 3 days after cell plating. The medium was changed 24 h later, after which the cells were washed 3 times. The conditioned medium was collected 2 days later. To test the seeding activity of the conditioned medium, the medium was transferred to primary neurons and α‐syn‐HEK293 cells and incubated for 10 days and 2 days, respectively. To observe the aggregation of α‐syn, the cells were fixed with 4% paraformaldehyde for 15 min at room temperature. The cells were permeabilized with 1% TX‐100 in PBS for 30 min at room temperature to eliminate soluble proteins.

### Proteinase K (PK) Digestion

Equal amounts of naïve PFFs and BV2‐α‐syn and α‐syn N103 PFFs were mixed with 2.5 µg mL^−1^ PK in PBS and incubated at 37 °C for 30 min. The reaction was stopped by the addition of 1 mM phenylmethylsulfonyl fluoride (PMSF). The samples were boiled in 1× loading buffer for 10 min. The bands of the PK digestion were detected by Coomassie blue staining.

### Membrane Protein Extraction

The membrane proteins were extracted using the Membrane and Cytosol Protein Extraction Kit. The cells were suspended in reagent A containing PMSF, incubated on ice for 15 min, and then freeze‐thawed 3 times in liquid nitrogen. After centrifugation at 700 × g for 10 min at 4 °C, the supernatant was collected and then centrifuged at 14 000 × g for 30 min at 4 °C to precipitate the cell membrane debris. The supernatant was collected as the cytoplasmic protein fraction. The pellets were further centrifuged at 14 000 × g for 30 s at 4 °C to remove the supernatant. The precipitates were resuspended in reagent B containing PMSF and then vigorously vortexed for 5 s and incubated on ice for 10 min. The vortexing and ice incubations were repeated 2 times. Finally, the samples were centrifuged at 14 000 × g for 10 min at 4 °C. The supernatant was collected as the cell membrane protein.

### Mass Spectrometry Analysis

LC‐MS/MS data acquisition was carried out on a Q Exactive Plus mass spectrometer coupled with an EASY‐nLC 1200 system (both Thermo Scientific). Peptides were picked up by an autosampler and transferred to a C18 analytical column (50 µm × 15 cm, 2 µm particle size, 100 Å pore size, Acclaim PepMap C18 column, Thermo) for separation. Mobile phase A (0.1% formic acid) and mobile phase B (80% ACN, 0.1% formic acid) were used to establish a 60 min gradient. A constant flow rate was set at 300 nL min^−1^. For DDA mode analysis, each scan cycle consisted of one full‐scan mass spectrum (R = 70 K, AGC = 3e^6^, max IT = 20 ms, scan range = 350–1800 m/z; R: resolution; AGC: automatic gain control; IT: injection time) followed by 15 MS/MS events (R = 17.5 K, AGC = 2e^5^, max IT = 50 ms). The 15 most abundant ions were subsequently detected with an isolation width of 1.6 Da and fragmented using HCD with a collision energy of 28. Former target ion exclusion was set for 35 s. MS raw data were analyzed with MaxQuant (V1.6.6) using the Andromeda database search algorithm.^[^ ^55^ ^]^ The spectral files were searched against the UniProt Human proteome database^[^ ^56^ ^]^ using the following parameters: LFQ mode was used for quantification; variable modifications, oxidation (M) & acetyl (Protein N‐term); fixed modifications, carbamidomethyl (C); digestion, trypsin/P; and matching between runs was used for identification transfer. Search results were filtered with a 1% false discovery rate (FDR) at both the protein and peptide levels. Peptides with at least 6 residues were selected.

### GST Pull‐Down and His Pull‐Down Assays

GST‐tagged TREM2 plasmids and FLAG‐TREM2 plasmids were transfected into HEK293 cells. The cells were collected 48 h after transduction, lysed in NP‐40 lysis buffer supplemented with protease and phosphatase inhibitor cocktail at 4 °C for 30 min and subsequently centrifuged at 15 000 rpm for 15 min at 4 °C. The supernatants were collected. For the GST pull‐down assay, cell lysates containing GST‐TREM2 were incubated with His‐α‐syn monomer or His‐α‐syn PFFs together with glutathione agarose overnight at 4 °C. Similarly, for the His pull‐down assay, His‐α‐syn monomer or His‐α‐syn PFFs were incubated with Ni‐NTA agarose FLAG‐tagged TREM2 supernatants were added to the beads and incubated overnight at 4 °C. The beads were washed 3 times with PBS and boiled in 1× SDS loading buffer. The proteins were analyzed by Western blotting.

### Preparation of TX100‐Soluble and ‐Insoluble Fractions from Cell Cultures

The BV2 cells were collected and resuspended in pre‐cooled Tris‐buffered saline (TBS) (50 mM Tris, 150 mM NaCl, pH 7.4) supplemented with 1% TX‐100 and a protease and phosphatase inhibitor cocktail. The samples were then sonicated and centrifuged at 100 000 × g for 30 min. The supernatant was isolated and labeled with the soluble TX‐100 fraction. The pellets were washed with TBS and centrifuged at 100 000 × g for 30 min at 4 °C. The pellet was re‐suspended in 2% SDS in TBS and termed the TX‐100‐insoluble fraction. The concentrations of all the samples were normalized using the BCA protein quantitation assay. The content of α‐syn in the TX‐100‐soluble and ‐insoluble fractions was further analyzed via Western blotting.

### Western Blot Analysis

The cells and mouse brain tissue were extracted in NP‐40 lysis buffer and RIPA buffer supplemented with protease and phosphatase inhibitor cocktail, respectively, followed by centrifugation at 15 000 rpm for 30 min at 4 °C. The total protein levels in the supernatants were quantified by the bicinchoninic acid assay before the samples were boiled in 1× loading buffer. After SDS‐PAGE, the proteins were transferred onto a nitrocellulose membrane. The membranes were incubated with 5% skim milk for 1 h at room temperature and incubated at 4 °C overnight with the appropriate primary antibodies: anti‐P2Y12 antibody (1:2000), anti‐MAP2 antibody (1:1000), anti‐GFAP antibody (1:1000), anti‐GAPDH antibody (1:10000), anti‐phospho‐α‐syn (Ser129) antibody (1:1000), anti‐tyrosine hydroxylase antibody (1:1000), anti‐α‐syn (Syn211) antibody (1:1000), anti‐FLAG antibody (1:2000), anti‐GST antibody (1:10000), anti‐α‐syn N103 antibody (1:2000). The membranes were washed for 1 h in TBST and incubated with HRP‐conjugated secondary antibodies at room temperature for 1 h. The signals were visualized using enhanced chemiluminescent (ECL) substrates.

### Immunohistochemistry and Immunofluorescence

The human brain sections and mouse brain paraffin‐embedded sections were deparaffinized in xylene and hydrated through descending ethanol. After being placed in antigen retrieval solution (0.1 M sodium citrate, pH 6.0) at 94 °C for 20 min, the sections were incubated in 3% hydrogen peroxide for 10 min to eliminate nonspecific background staining caused by endogenous peroxidase activity. After blocking in 3% BSA and 0.3% Triton X‐100 for 30 min, the sections were incubated at 4 °C overnight with the following primary antibodies: anti‐Iba1 (1:1000), anti‐MAP2 (1:1000), anti‐GFAP (1:500), anti‐phospho‐α‐syn (Ser129) (1:500), and anti‐tyrosine hydroxylase (1:1000) antibodies. The signals were detected according to the manufacturer's instructions for the High‐Efficiency IHC Detection System Kit (Absin, abs957). For the tissue immunofluorescence staining experiment, a mixture of Alexa Fluor 488‐ and Alexa Fluor 594‐conjugated secondary antibodies was used after primary antibody incubation. For immunofluorescence staining of the cell cultures, the cells were fixed with 4% paraformaldehyde and 0.1% Triton X‐100 for 15 min at room temperature. The cells were washed with PBS, blocked with 3% BSA for 30 min, and incubated overnight at 4 °C with primary antibodies. The cells were then washed with PBS and incubated with Alexa Fluor 488‐ and Alexa Fluor 594‐conjugated secondary antibodies for 2 h at room temperature in the dark. The cell nuclei were stained with DAPI.

### Behavioral Analysis

The motor function of the wild‐type, TREM2 KO, and AEP KO mice was evaluated by performing the tail suspension test, rotarod test, grid performance test, balance beam test, pole test, and footprint test. The tail suspension test was conducted, and the limb clasp score was assessed as previously described.^[^ ^57^, ^58^ ^]^ Mice were observed by hanging their tails in front of a white wall and monitoring when they were immobile. The posture of the mice was quantified as follows: 0, no limb clasping; 1, one hind limb exhibits incomplete splay and loss of mobility, toes exhibit normal splay; 2, both hind limbs exhibit incomplete splay and loss of mobility, toes exhibit normal splay; 3, both hind limbs exhibit clasping with curled toes and immobility; 4, forelimbs and hind limbs exhibit clasping and are crossed, curled toes and immobility. In the rotarod test, mice were placed on a spinning rod at a gradually accelerating spinning rate from 5 to 40 r.p.m. within 3 min to assess locomotion activity. Before being subjected to the formal test, the mice were trained 3 times a day for 3 consecutive days, and a 30‐min break was given between trial intervals. The latency between the start of the rod and the fall of the mice was recorded. In the grid performance test, the mice were placed on a grid (1.5 m × 1.5 m with 5 mm diameter) and inverted after being shaken horizontally several times. Once the grid was flipped, the mice were hung upside down by their claws until they fell to the ground. The intervals to drop were recorded. In the balance beam test, we applied two 80 cm beams with flat widths of 1.6 cm and 0.9 cm. The 1.6 cm wide beams were used for training, and the mice were trained 3 times a day for 2 consecutive days. The test was performed on the third day on 0.9 cm wide beams. The latency at which the mice crossed the center of the beam by 50 cm was recorded. In the pole test, mice were placed head‐upward on the top of a vertical rough‐surfaced wooden pole (45 cm length with 1 cm diameter) and allowed to autonomously descend. The formal test was performed after the mice were trained 3 times a day for 2 consecutive days. The intervals from the descent of the mice until they reached the midpoint of the pole and until their claws touched the floor were recorded. In the footprint test, the front claws were colored red, and the hind claws were colored blue. The step length and step width were recorded in this study.

### High‐Performance Liquid Chromatography Analysis

The striatal tissues were separated and rapidly frozen in liquid nitrogen. Before the test, the tissues were weighed and homogenized in 0.1 M perchloric acid solution containing 0.1% cysteine and then rapidly centrifuged at 14 000 rpm for 15 min at 4 °C. The supernatant fractions were filtered and injected into a chromatographic column (4.6 mm × 150 mm) and separated by a mobile phase (8% methanol in 3 mM sodium heptane sulfonate, 0.2 mM EDTA, 100 mM sodium acetate, 85 mM citric acid) at a flow rate of 1 mL min^−1^. The concentrations of DA, DOPAC, and HVA were quantified via comparison to the standard curve in this study.

### Single‐Cell RNA Sequencing Analysis

The open access single‐cell RNA sequencing data were obtained from a previous study^[^ ^32^ ^]^ and analyzed with the help of Singleron Biotechnologies. Scanpy v1.8.1 was used for quality control, dimensionality reduction, and clustering with Python 3.7. For each sample dataset, we filtered the expression matrix by the following criteria: 1) cells with gene count less than 200 or with top 2% gene count were excluded; 2) cells with top 2% UMI count were excluded; 3) cells with mitochondrial content > 5% were excluded; and 4) genes expressed in less than 5 cells were excluded. After filtering, 46 017 cells were retained for the downstream analyses, with an average of 2221 genes and 3916 UMIs per cell. The raw count matrix was normalized by total counts per cell and logarithmically transformed into a normalized data matrix. The top 2000 variable genes were selected by setting flavor = “seurat”. Principal component analysis (PCA) was performed on the scaled variable gene matrix. The top 20 principal components were used for clustering and dimensional reduction. Cell clusters were visualized by using uniform manifold approximation and projection (UMAP).^[^ ^59^ ^]^ The cell type identification of each cluster was determined according to the expression of canonical markers from the reference database SynEcoSysTM (Singleron Biotechnology). SynEcoSysTM contains collections of canonical cell type markers for single‐cell seq data from CellMakerDB, PanglaoDB, and recently published literature. To obtain a high‐resolution map of microglia and astrocytes, cells from the specific cluster were extracted and reclustered for more detailed analysis following the same procedures described above. Gene set scoring was performed using the R package UCell v 1.1.0.^[^ ^60^ ^]^ UCell scores are based on the Mann‐Whitney U statistic by ranking query genes in order of their expression levels in individual cells.

### Counting of Dopaminergic Neurons

For dopaminergic neuron counting, the entire substantia nigra region of each mouse brain was sliced into consecutive 4 µm‐thick paraffin sections. Every 6th section was involved in the counting procedure covering the entire extent of the substantia nigra region for TH‐positive cells in the ipsilateral SNpc region. The cell counts were calculated based on the sum of TH‐positive cells in all involved mouse brain sections.

### Optical Densitometry Analysis

The number of TH‐positive terminals in the striatum was measured according to a procedure described previously.^[^ ^61^ ^]^ The optical density of the entire striatum containing the 6 coronal layers at equal distances relative to bregma was measured using ImageJ software (version 2.1.0/1.53c) and the IHC Profiler plug‐in according to the user manual.

### Quantification and Statistical Analysis

All data were expressed as the mean ± s.e.m. (standard error of the mean) from three or more independent experiments and analyzed with GraphPad Prism software (version 8.0). Statistical analysis was performed using unpaired two‐tailed Student's *t*‐test for two‐group comparisons. When variances were not equal or the data were ranked, the Mann‐Whitney test was applied. One‐way ANOVA was applied to confirm the significant main effects and differences among three or more groups followed by Tukey's multiple comparisons for *post hoc* tests. For the grouped data, two‐way ANOVA was used to analyze the levels of significant main effects followed by Bonferroni's multiple comparisons for *post hoc* tests. The sample size was determined based on primary results or similar experiments conducted in previous studies. Differences with *P* values less than 0.05 were considered significant and were presented as *p < 0.05, **p < 0.01, ***p < 0.001.

## Conflict of Interest

The authors declare no conflict of interest.

## Author Contributions

M.X. and D.X. contributed equally to this work. Z.Z. performed conceptualization. H.Y., L.M., and X.Z. performed methodology. M.X. and D.X. performed investigation. M.X. performed writing – original draft. Z.Z. and L.M. performed funding acquisition. J.C., Y.T., and X.Y. performed resources. X.N., S.N., Z.Z., C.L., Q.C., and K.Y. performed supervision.

## Supporting information



Supporting Information

## Data Availability

The data that support the findings of this study are available from the corresponding author upon reasonable request.
