# Supplementary material for: Microglia Process α‐Synuclein Fibrils and Enhance their Pathogenicity in a TREM2‐Dependent Manner
Source: Adv Sci (Weinh). 2024 Dec 12;12(7):2413451. doi: 10.1002/advs.202413451 (PMC11831461; doi:10.1002/advs.202413451)
Supplement: Supplementary file 1 — Supporting Information [file ADVS-12-2413451-s001.docx]

Supporting Information

**Microglia process α-synuclein fibrils and enhance their pathogenicity in a TREM2-dependent manner**

Min Xiong, Danhao Xia, Honglu Yu, Lanxia Meng, Xingyu Zhang, Jiehui Chen, Ye Tian, Xin Yuan, Xuan Niu, Shuke Nie, Zhaohui Zhang, Chaoyang Liu, Qiang Chen, Keqiang Ye, Zhentao Zhang*


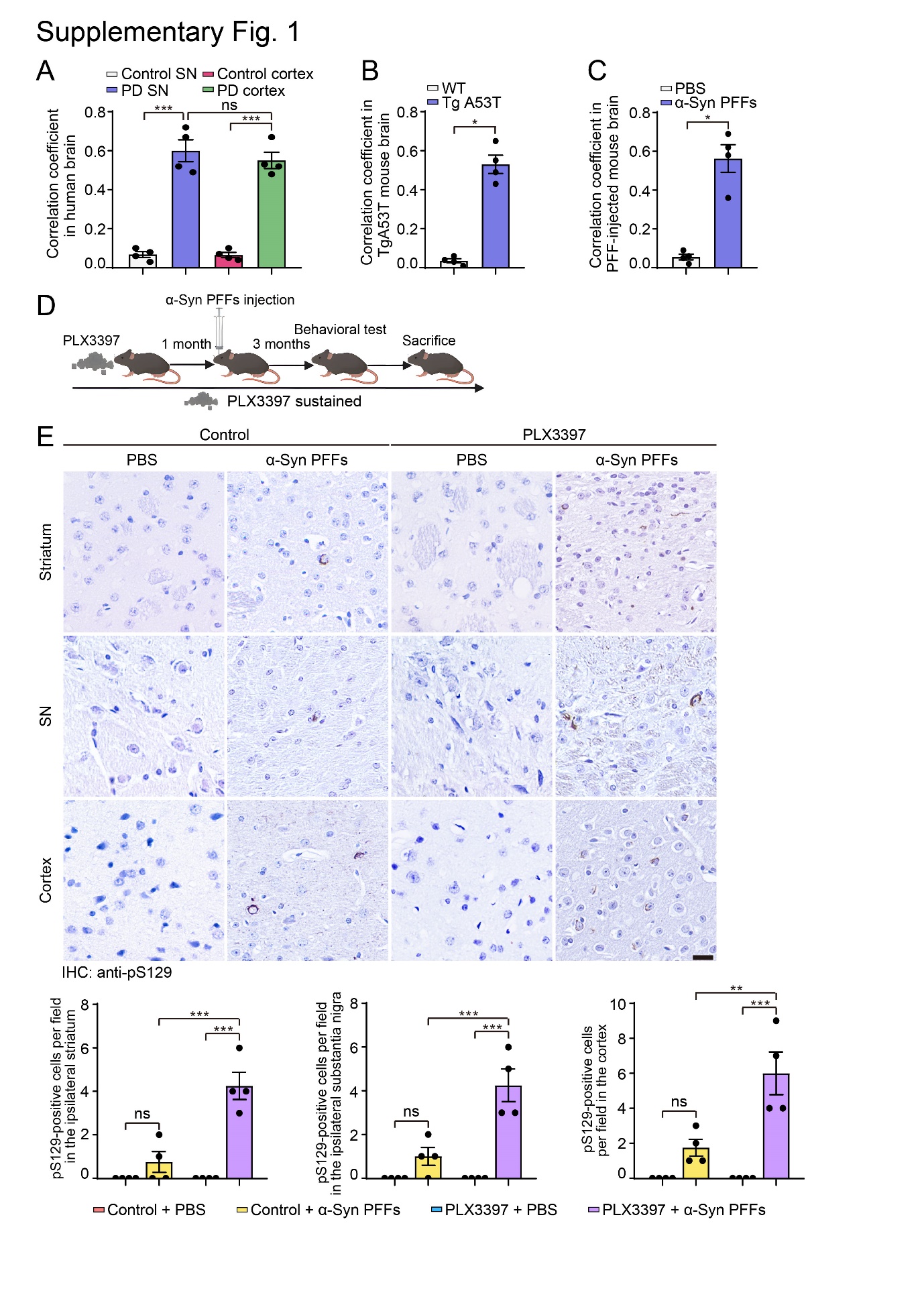


**Supplementary Figure 1. PLX3397 treatment slightly aggravates α-syn pathology at 1 mpi. Related to Figure 1.**

(A-C) Quantitation of the colocalization of Iba1 and pS129 in PD patients (A), A53T mice (B) and α-syn PFF-injected mice (C) (mean ± s.e.m.; n = 4 mice per group; **P* < 0.05; ****P* < 0.001; one-way ANOVA; Mann-Whitney test). ns, not significant. (D) Schematic diagram of mouse treatment. The mice were treated with PLX3397 for 1 month, followed by intrastriatal α-syn PFF injection. PLX3397 was administered at 290 mg/kg/d throughout the experiment. (E) Representative images of α-syn pathology in mice treated with PLX3397 for 1 month (mean ± s.e.m.; n = 4 mice per group; ***P* < 0.01; ****P* < 0.001; two-way ANOVA). Scale bar, 20 μm. ns, not significant.


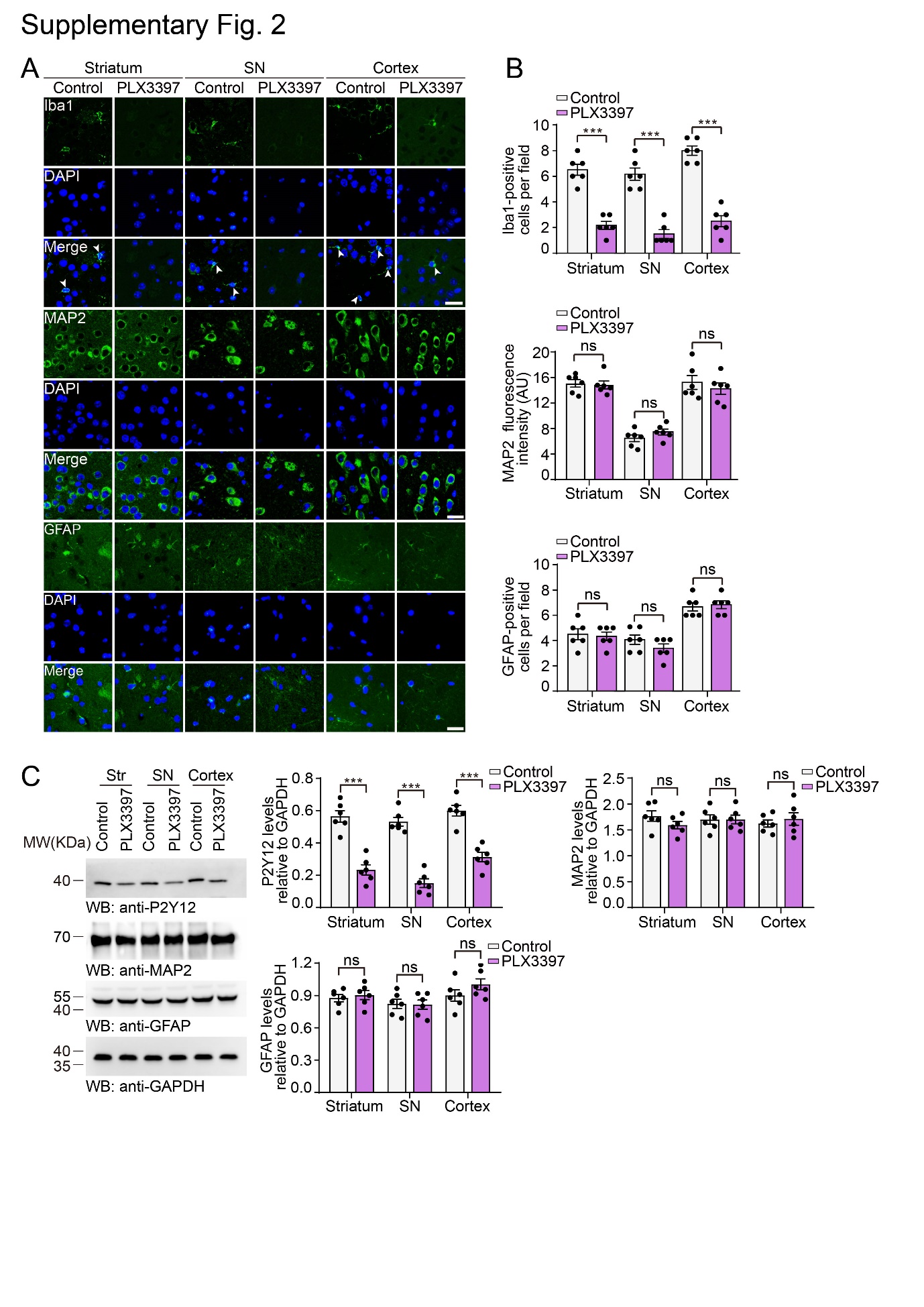


**Supplementary Figure 2. PLX3397 depletes microglia in the mouse brain. Related to Figure 1.**

(A-B) Immunofluorescence showing the density of Iba1- and GFAP-positive cells and the MAP2 fluorescence intensity in the striatum, substantia nigra, and cortex 1 month after PLX3397 treatment (mean ± s.e.m.; n = 6 mice per group; ****P* < 0.001; *t*-test). Iba1/MAP2/GFAP, green; DAPI, blue. Scale bar, 20 μm. ns, not significant. (C) Western blots showing the levels of P2Y12, MAP2, and GFAP in the striatum, substantia nigra, and cortex of mice 1 month after PLX3397 treatment (mean ± s.e.m.; n = 6 mice per group; ****P* < 0.001; two-way ANOVA). ns, not significant.


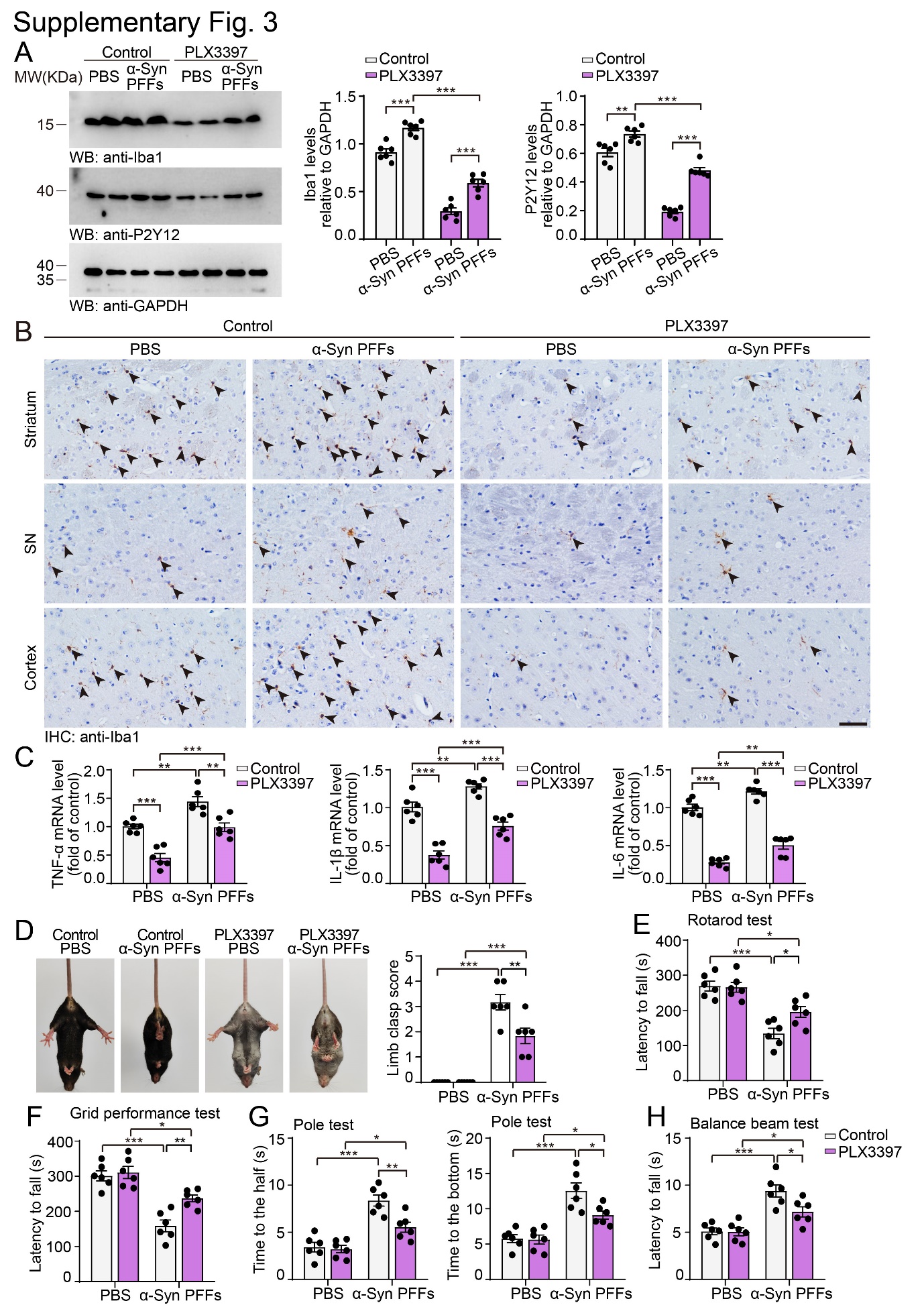


**Supplementary Figure 3. Depletion of microglia partially attenuates motor impairment induced by α-syn PFFs. Related to Figure 1.**

(A) Western blot analysis revealing the levels of Iba1 and P2Y12 in the striatum at 3 mpi (mean ± s.e.m.; n = 6 mice per group; ***P* < 0.01; ****P* < 0.001; two-way ANOVA). (B) Representative images of microglial intensity at 3 mpi. Scale bar, 50 μm. (C) RT-PCR analysis of the mRNA levels of TNF-α, IL-1β and IL-6 in the striatum at 3 mpi (mean ± s.e.m.; n = 6 mice per group; ***P* < 0.01; ****P* < 0.001; two-way ANOVA). (D) Representative images of the tail suspension test at 3 mpi (mean ± s.e.m.; n = 6 mice per group; ***P* < 0.01; ****P* < 0.001; two-way ANOVA). Treatment with PLX3397 induced fur depigmentation due to c-kit inhibition, as reported previously.^[1]^ (E-H) The rotarod test, grid performance test, pole test, and balance beam test were performed at 3 mpi (mean ± s.e.m.; n = 6 mice per group; **P* < 0.05; ***P* < 0.01; ****P* < 0.001; two-way ANOVA). ns, not significant.


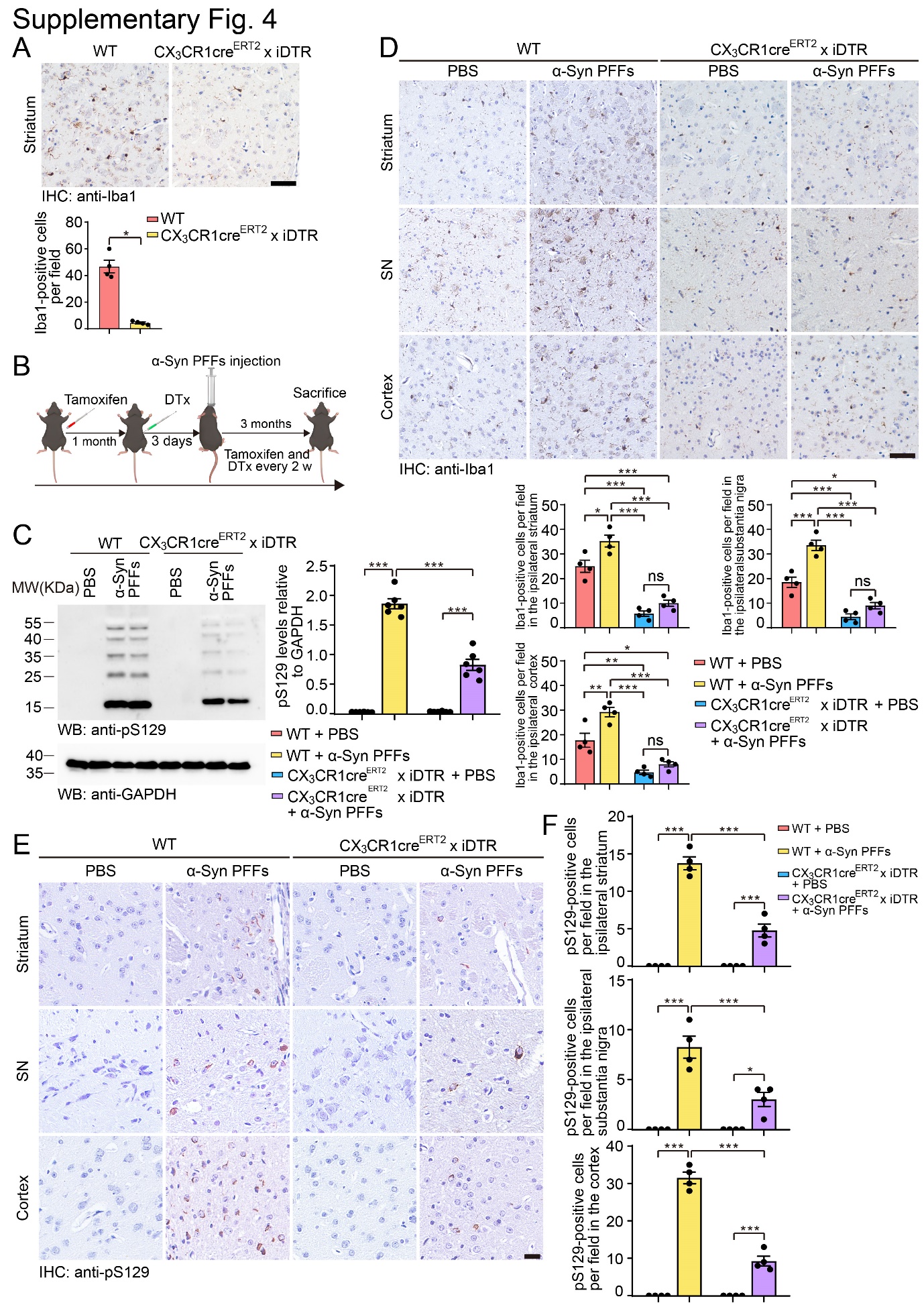


**Supplementary Figure 4. Microglial depletion alleviates α-syn pathology in CX_3_CR1cre^ERT2^ × iDTR mice. Related to Figure 1.**

(A) Quantification of microglia at 1 day after the administration of diphtheria toxin (DTx). (mean ± s.e.m.; n = 4 mice per group; **P* < 0.05; Mann-Whitney test). Scale bar, 50 μm. (B) Experimental timeline for microglial depletion, α-syn PFF injection, and pS129 pathology analysis in wild-type and CX_3_CR1cre^ERT2^ × iDTR mice. (C) Western blot analysis revealing the levels of pS129 in the striatum at 3 mpi (mean ± s.e.m.; n = 6 mice per group; ****P* < 0.001; two-way ANOVA). (D) Immunohistochemistry of Iba1 in the striatum, substantia nigra, and cortex of CX_3_CR1cre^ERT2^ × iDTR mice at 3 mpi (mean ± s.e.m.; n = 6 mice per group; **P* < 0.05; ***P* < 0.01; ****P* < 0.001; two-way ANOVA). Scale bar, 50 μm. ns, not significant. (E, F) Representative images and quantification of α-syn pathology in CX_3_CR1cre^ERT2^ × iDTR mice at 3 mpi (mean ± s.e.m.; n = 4 mice per group; **P* < 0.05; ****P* < 0.001; two-way ANOVA). Scale bar, 20 μm.


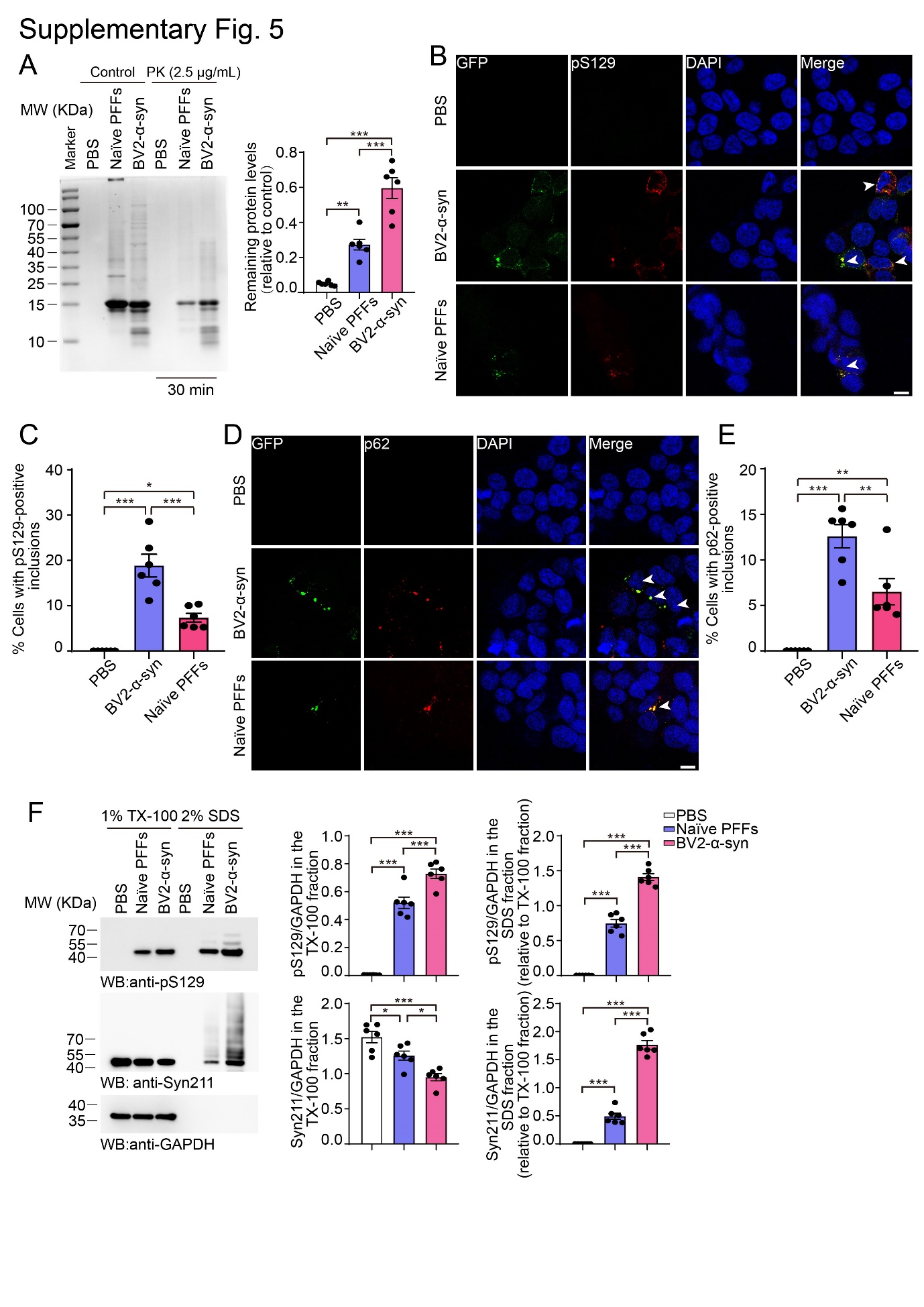


**Supplementary Figure 5.** **BV2-processed α-syn PFFs show enhanced seeding activity. Related to Figure 2.**

(A) Proteinase K (PK) digestion assay. Equal amounts of naïve PFFs and BV2-processed α-syn PFFs were incubated with 2.5 μg/mL PK for 30 min and then analyzed by Coomassie blue staining (mean ± s.e.m.; n = 6 independent experiments; ***P* < 0.01; ****P* < 0.001; one-way ANOVA). (B-E) Insoluble α-syn aggregates induced by BV2-processed α-syn species and naïve α-syn PFFs in α-syn-HEK293 cells. The cells were treated with 1% Triton X-100 to eliminate soluble α-syn. The insoluble aggregates colocalize with pS129 (B, C) and p62 (D, E) (mean ± s.e.m.; n = 6 independent experiments; **P* < 0.05; ***P* < 0.01; ****P* < 0.001; one-way ANOVA). α-Syn aggregates, green; pS129/p62, red; DAPI, blue. Scale bar, 10 μm. (F) Sequential extraction assay showing the TX-100-soluble and -insoluble α-syn species in α-syn-HEK293 cells transduced with naïve PFFs or BV2-processed α-syn PFFs (mean ± s.e.m.; n = 6 independent experiments; **P* < 0.05; ****P* < 0.001; one-way ANOVA).


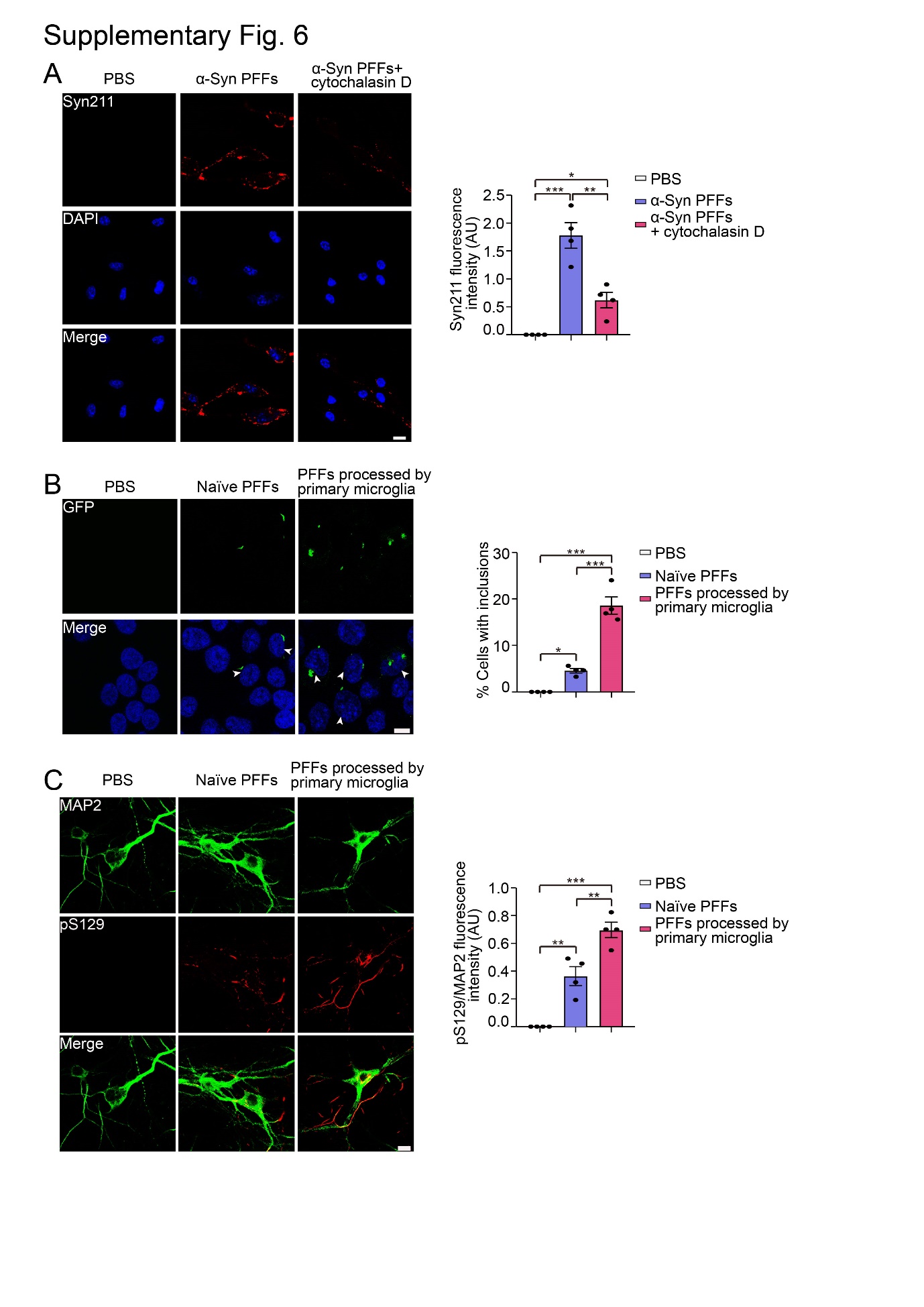


**Supplementary Figure 6. Primary microglia phagocytose and process α-syn. Related to Figure 2.**

(A) Immunofluorescence showing the uptake of α-syn PFFs by primary microglia in the presence or absence of cytochalasin D (mean ± s.e.m.; n = 4 independent experiments; **P* < 0.05; ***P* < 0.01; ****P* < 0.001; one-way ANOVA). Syn211, red; DAPI, blue. Scale bar, 10 μm. (B) Insoluble α-syn aggregates in α-syn-HEK293 cells transduced with conditioned medium from α-syn PFF-treated primary microglia or naïve α-syn PFFs. The cells were treated with 1% Triton X-100 to eliminate soluble α-syn (mean ± s.e.m.; n = 4 independent experiments; **P* < 0.05; ****P* < 0.001; one-way ANOVA). α-Syn aggregates, green; DAPI, blue. Scale bar, 10 μm. (C) Phosphorylation of α-syn in neurons transduced with conditioned medium from PFF-treated primary microglia and naïve α-syn PFFs. The amount of α-syn species was normalized using ELISA (mean ± s.e.m.; n = 4 independent experiments; ***P* < 0.01; ****P* < 0.001; one-way ANOVA). α-Syn aggregates, red; MAP2, green; DAPI, blue. Scale bar, 10 μm. AU, arbitrary unit.


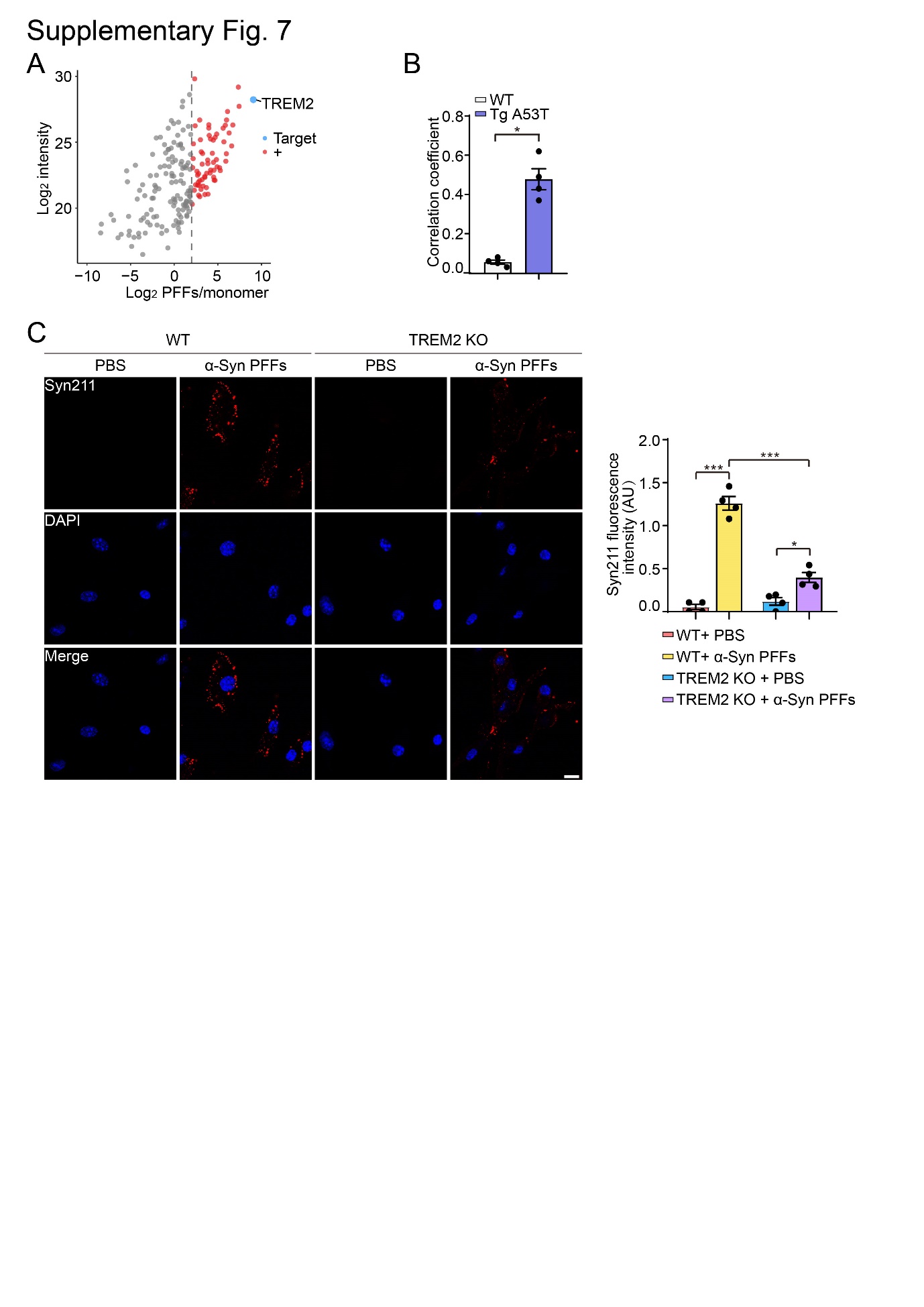


**Supplementary Figure 7. TREM2 mediates α-syn phagocytosis. Related to Figure 3.**

(A) The volcano map showing the intensity of the interaction between TREM2 and α-syn PFFs. (B) Quantitation of the colocalization of TREM2 with pS129 in the TgA53T mouse brain, as shown in Figure 3C (mean ± s.e.m.; n = 4 independent experiments; **P* < 0.05; Mann-Whitney test). (C) Immunofluorescence showing the uptake of α-syn PFFs by primary microglia from wild-type and TREM2 KO mice (mean ± s.e.m.; n = 4 independent experiments; **P* < 0.05; ****P* < 0.001; two-way ANOVA). Syn211, red; DAPI, blue. Scale bar, 10 μm.


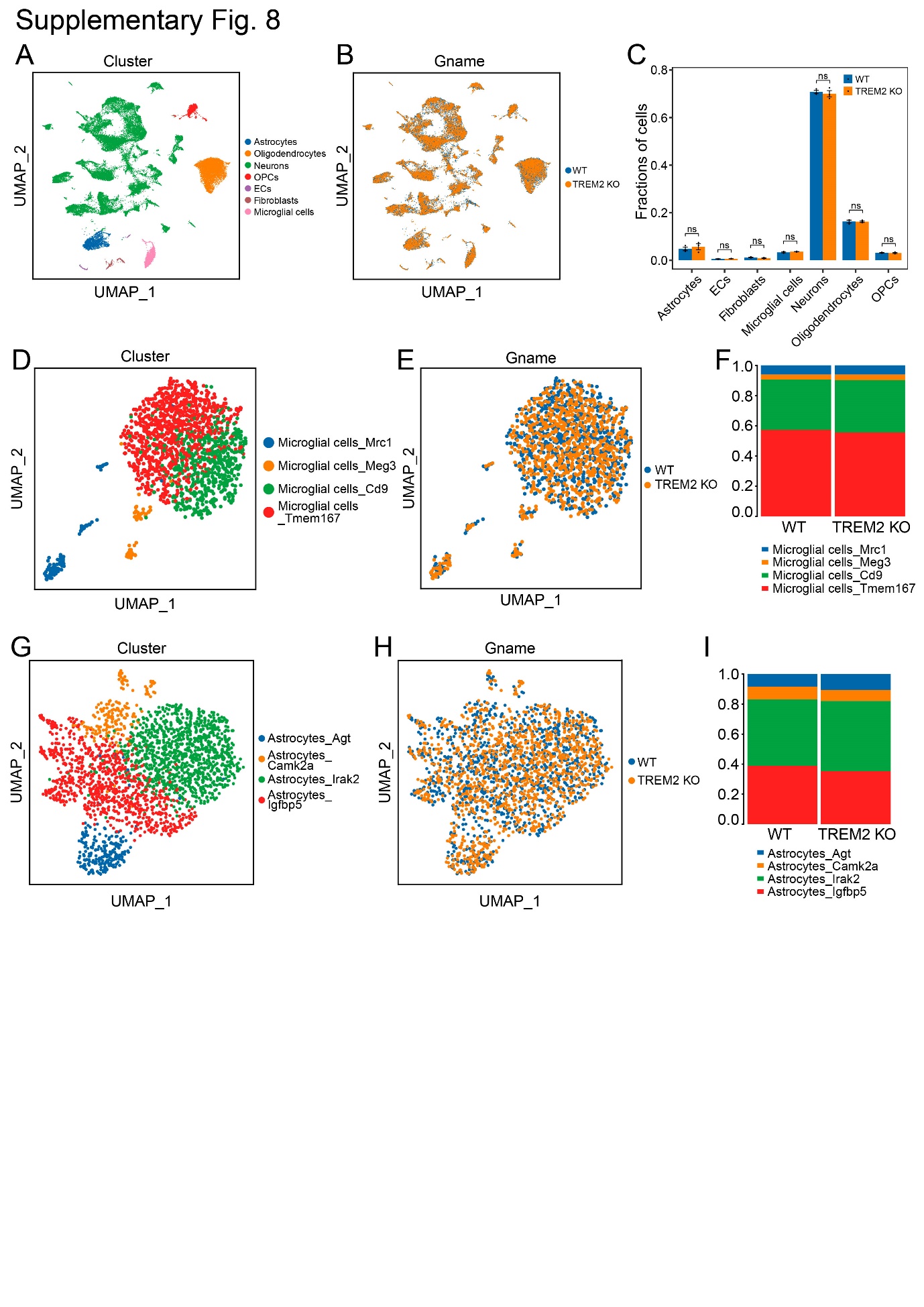


**Supplementary Figure 8. Single-cell RNA sequencing analysis of WT and TREM2 KO mice. Related to Figure 4.**

(A) Uniform manifold approximation and projection (UMAP) plot analyzed by 7 cell clusters, including astrocytes, oligodendrocytes, neurons, oligodendrocyte progenitor cells (OPCs), endothelial cells (ECs), fibroblasts, and microglia. (B) UMAP of the 7 cell clusters stained by the two groups. (C) Histogram showing the fraction of cells within 7 cell clusters in WT and TREM2 KO mice. (D) UMAP plot analyzed by 4 cell subclusters, including microglia_Mrc1, microglia_Meg3, microglia_Cd9, and microglia_Tmem167. (E) UMAP of the 4 cell subclusters stained by the two groups. (F) Stacked bar graph showing the proportions of 4 subclusters in the WT and TREM2 KO groups. (G) UMAP plot of 4 cell subclusters, including astrocytes_Agt, astrocytes_Camk2a, astrocytes_Irak2, and astrocytes_Igfbp5. (H) MAP of the 4 cell subclusters was stained by the two groups. (I) Stacked bar graph showing the proportions of 4 subclusters in WT and TREM2 KO mice.


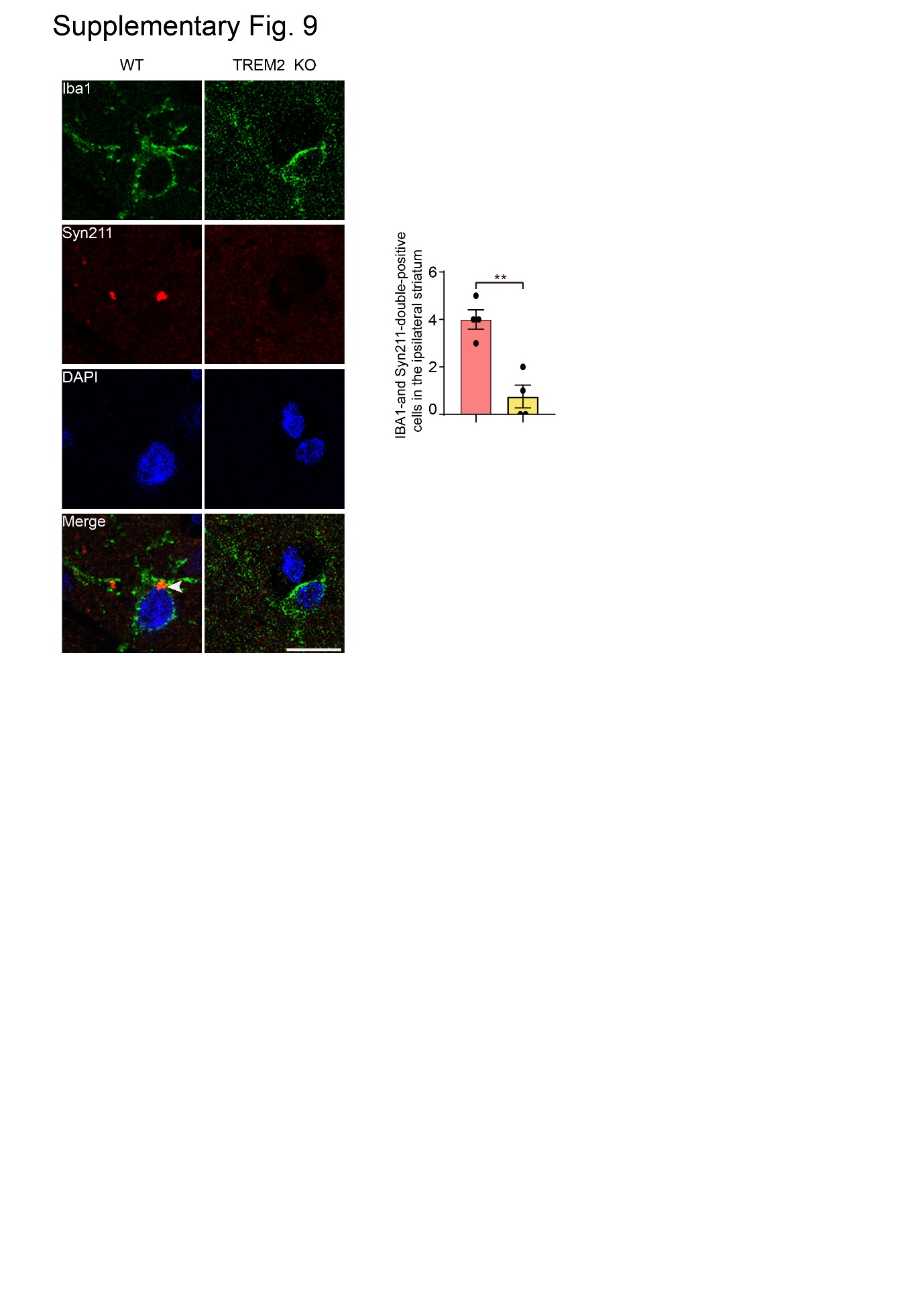


**Supplementary Figure 9. TREM2 mediates the phagocytosis of α-syn PFFs by microglia *in vivo*. Related to Figure 4.**

Double immunofluorescence staining for Iba1 and human α-syn in wild-type and TREM2 KO mice injected with α-syn PFFs. The sections were stained 3 days after injection (mean ± s.e.m.; n = 4 mice per group; ***P* < 0.01; *t*-test). Iba1, green; Syn11, red; DAPI, blue. Scale bar, 10 μm.


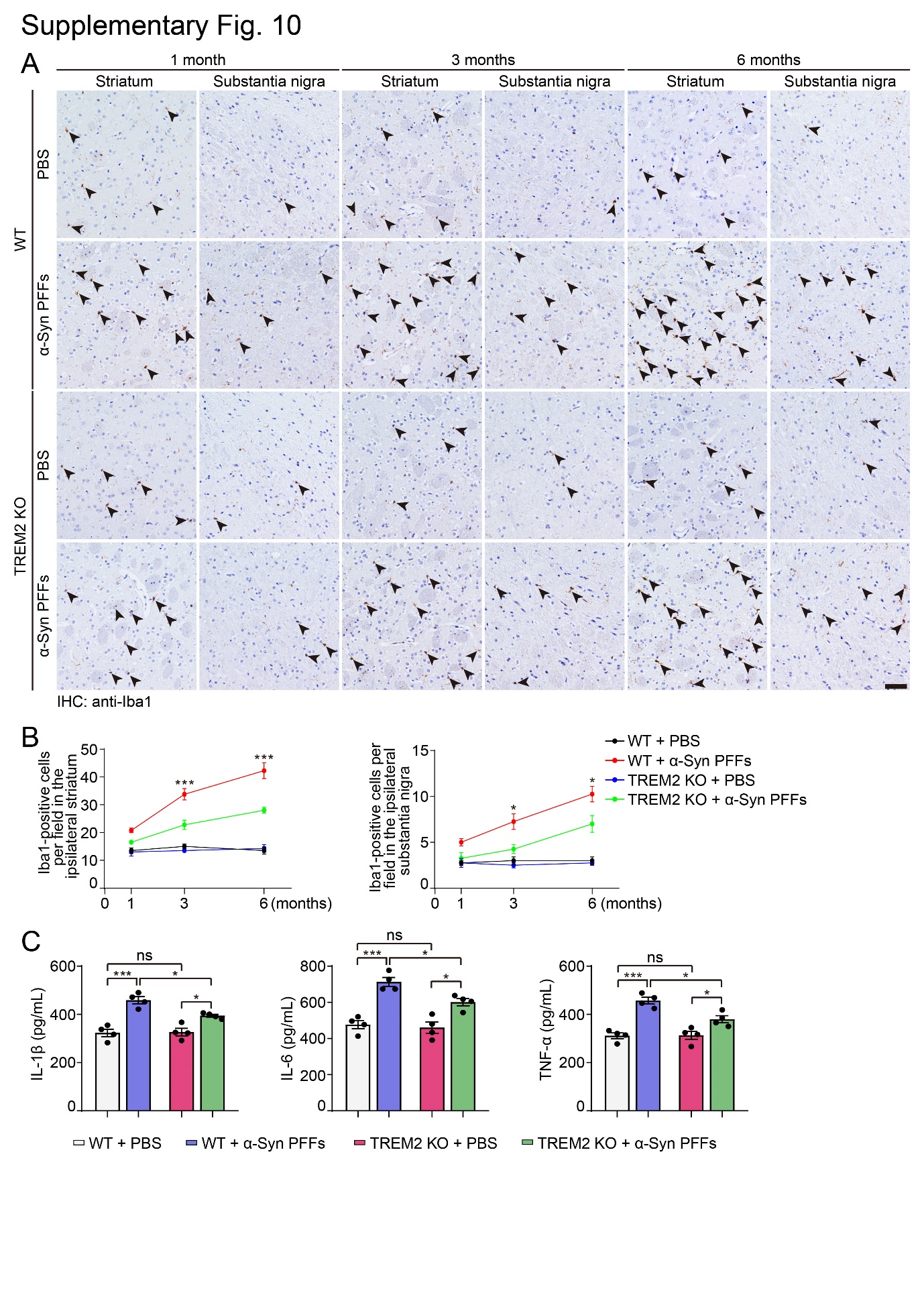


**Supplementary Figure 10. Knockout of TREM2 attenuates microglial activation induced by α-PFFs. Related to Figure 4.**

(A) Representative images of microglia in PFF-injected wild-type mice and TREM2 KO mice. Scale bar, 50 μm. (B) Quantification of Iba1-positive signals in the ipsilateral striatum and substantia nigra (mean ± s.e.m.; n = 4 mice per group; *P < 0.05; ***P < 0.001; two-way ANOVA). (C) ELISA quantification of IL-1β, IL-6, and TNF-α in brain lysates from wild-type or TREM2 KO mice treated with PBS or α-syn PFFs at 6 mpi (mean ± s.e.m.; n = 4 mice per group; **P* < 0.05; ****P* < 0.001; two-way ANOVA). ns, not significant.


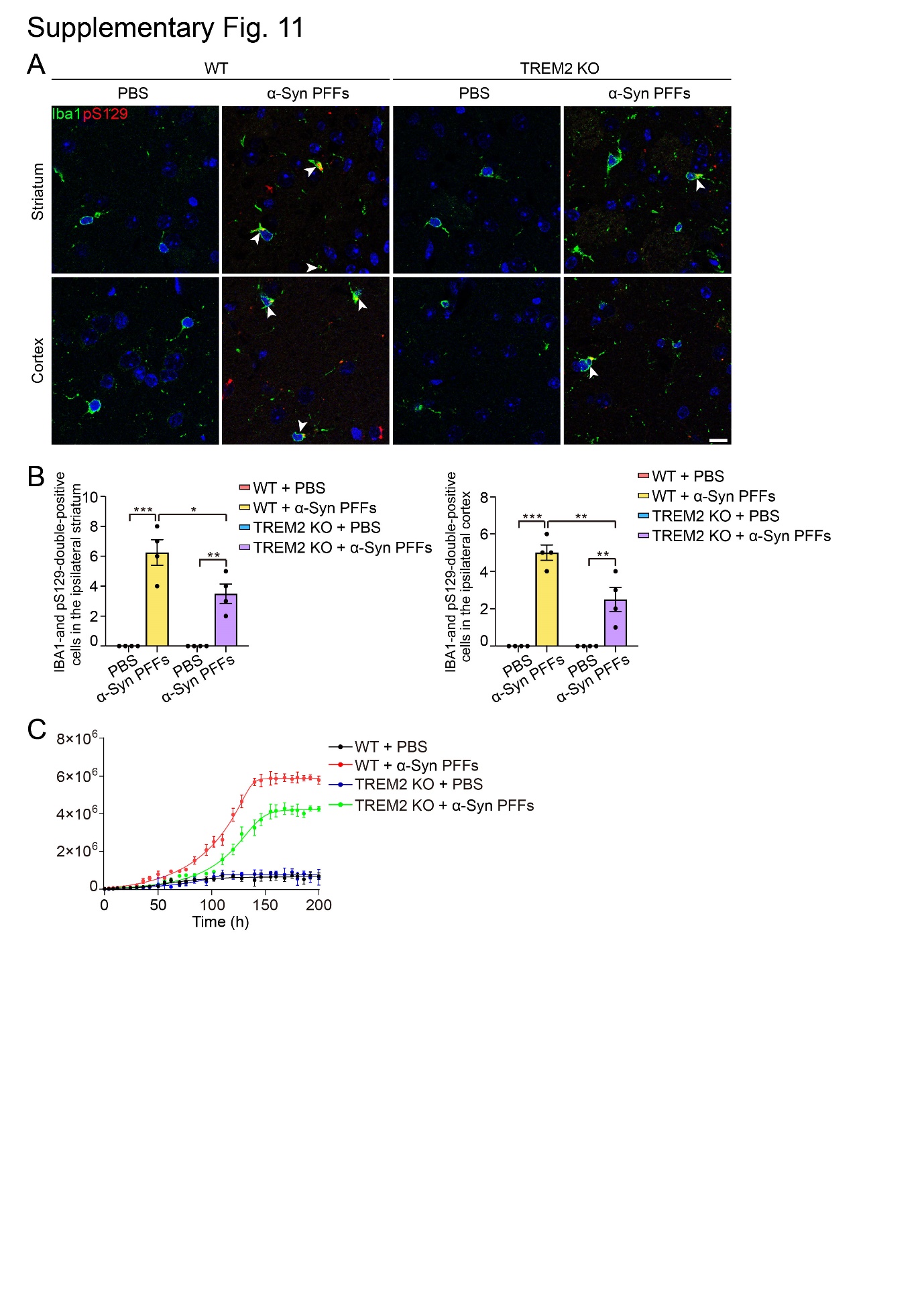


**Supplementary Figure 11.** **Deletion of TREM2 partially attenuates α-syn pathology *in vivo*. Related to Figure 4.**

(A) Immunofluorescence of Iba1 and pS129 in the striatum and cortex of mice injected with PBS and α-syn PFFs at 6 mpi. Iba1, green; pS129, red; DAPI, blue. Scale bar, 10 μm. (B) The intensity of Iba1- and pS129-double-positive cells (mean ± s.e.m.; n = 4 mice per group; **P* < 0.05; ***P* < 0.01; ****P* < 0.001; two-way ANOVA). (C) The aggregation kinetics of α-syn (1 mg/mL) fibrillization seeded by brain lysates from wild-type or TREM2 KO mice injected with α-syn PFFs. AFU: arbitrary fluorescence unit.


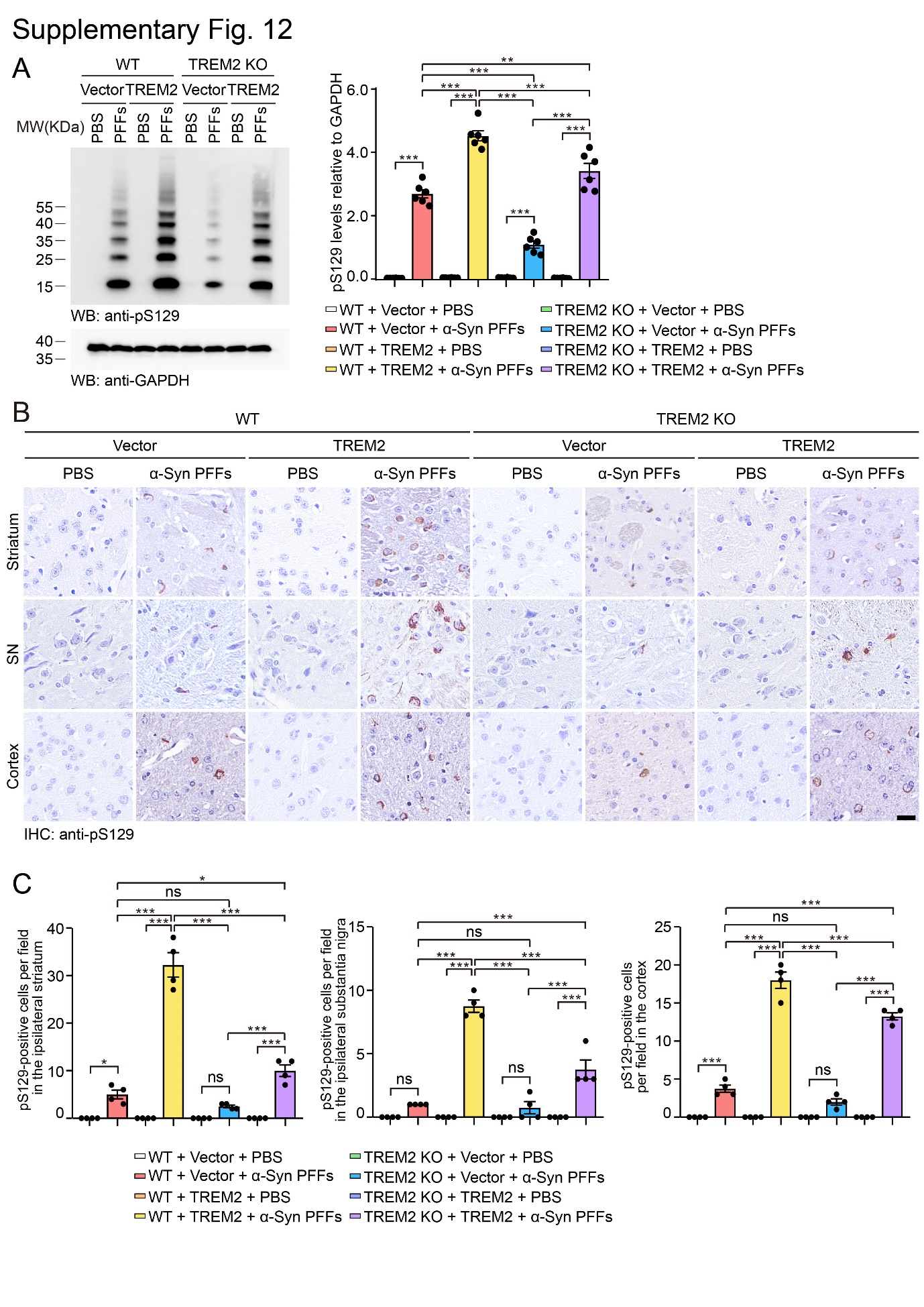


**Supplementary Figure 12.** **TREM2 overexpression aggravates α-syn pathology. Related to Figure 4.**

α-Syn PFFs together with AAV-TREM2 or control virus were injected into the striatum of wild-type and TREM2 KO mice. α-Syn pathology was determined at 3 mpi. (A) Western blots showing the levels of pS129 in the striatum (mean ± s.e.m.; n = 6 mice per group; ***P* < 0.01; ****P* < 0.001; one-way ANOVA). (B, C) Representative images and quantification of α-syn pathology at 3 mpi (mean ± s.e.m.; n = 4 mice per group; **P* < 0.05; ****P* < 0.001; one-way ANOVA). Scale bar, 20 μm. ns, not significant.


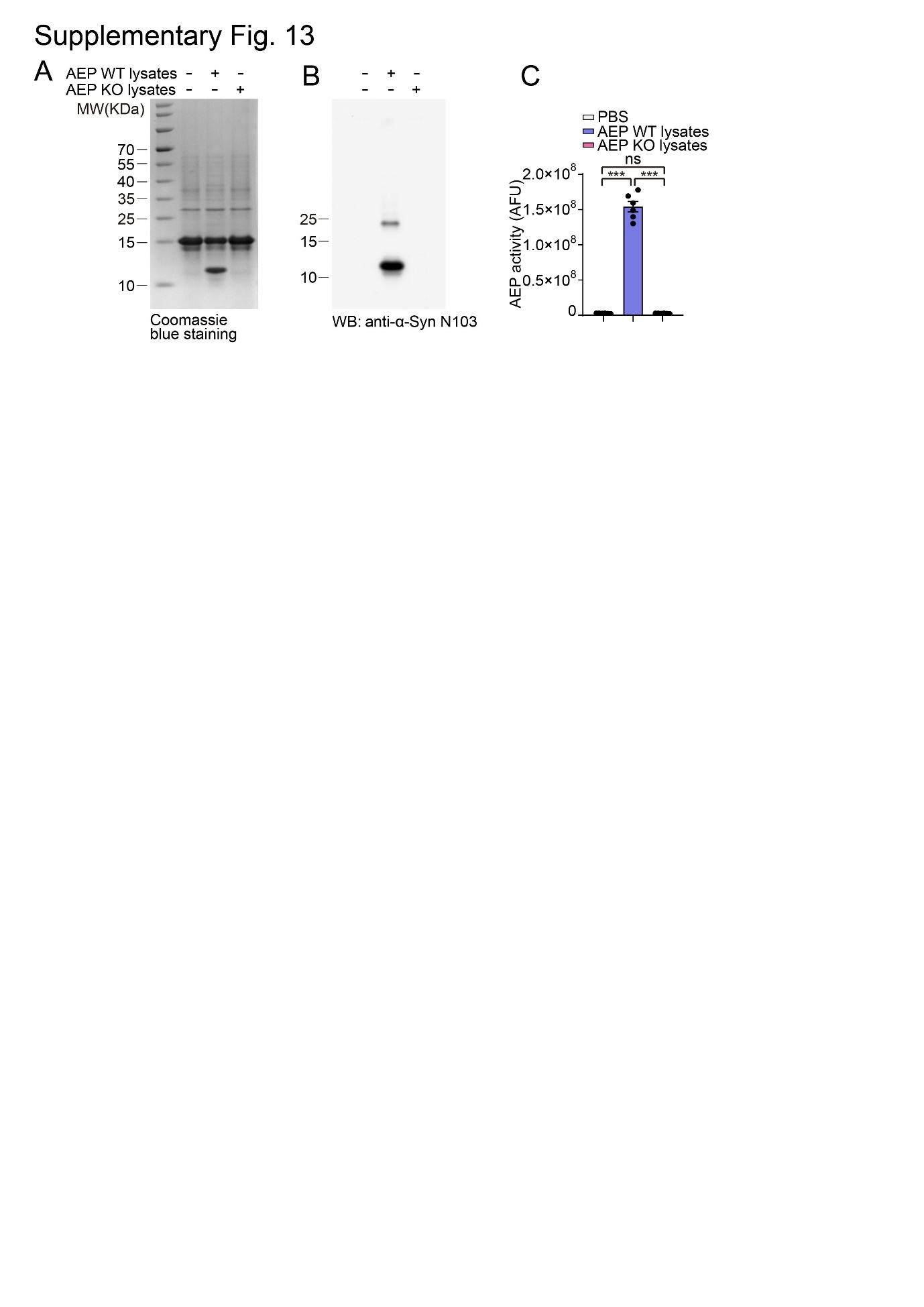


**Supplementary Figure 13. AEP generates α-syn N103 fragments. Related to Figure 6.**

(A, B) Coomassie blue staining (A) and Western blots (B) showing the generation of α-Syn N103 after α-Syn PFFs were incubated with brain lysates from wild-type and AEP KO mice. (C) AEP activity assay (mean ± s.e.m.; n = 6 independent experiments; ****P* < 0.001; one-way ANOVA). ns, not significant.


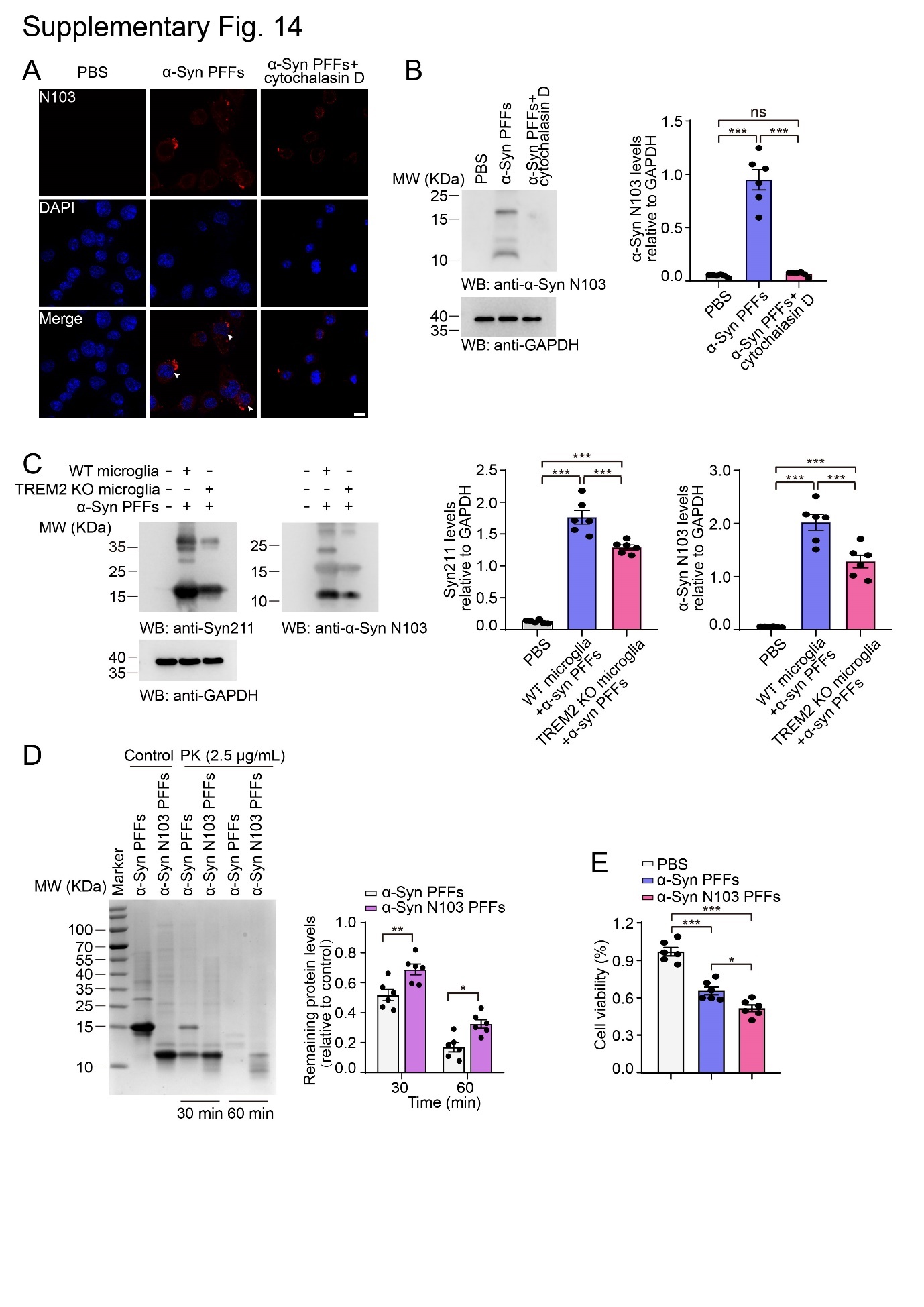


**Supplementary Figure 14.** **AEP mediates the cleavage of α-syn PFFs in microglia. Related to Figure 6.**

(A) Immunofluorescence showing the presence of N103 in BV2 cells after treatment with α-syn PFFs in the presence or absence of cytochalasin D. N103, red; DAPI, blue. Scale bar, 10 μm. (B) Western blot analysis showing the levels of N103 in BV2 cells treated with α-syn PFFs in the presence or absence of cytochalasin D (mean ± s.e.m.; n = 6 independent experiments; ****P* < 0.001; one-way ANOVA). ns, not significant. (C) Western blot analysis revealing the levels of α-syn and α-syn N103 fragments in primary microglia from wild-type and TREM2 KO mice treated with α-syn PFFs (mean ± s.e.m.; n = 6 independent per group; ****P* < 0.001; one-way ANOVA). (D) Proteinase K (PK) digestion assay. Equal amounts of α-syn PFFs and α-syn N103 PFFs were incubated with 2.5 μg/mL PK for 30 min and then analyzed by Coomassie blue staining (mean ± s.e.m.; n = 6 independent experiments; **P* < 0.05; ***P* < 0.01; two-way ANOVA). (E) Cell viability of neurons treated with α-syn PFFs and α-syn N103 PFFs (mean ± s.e.m.; n = 6 independent experiments; **P* < 0.05; ****P* < 0.001; one-way ANOVA).


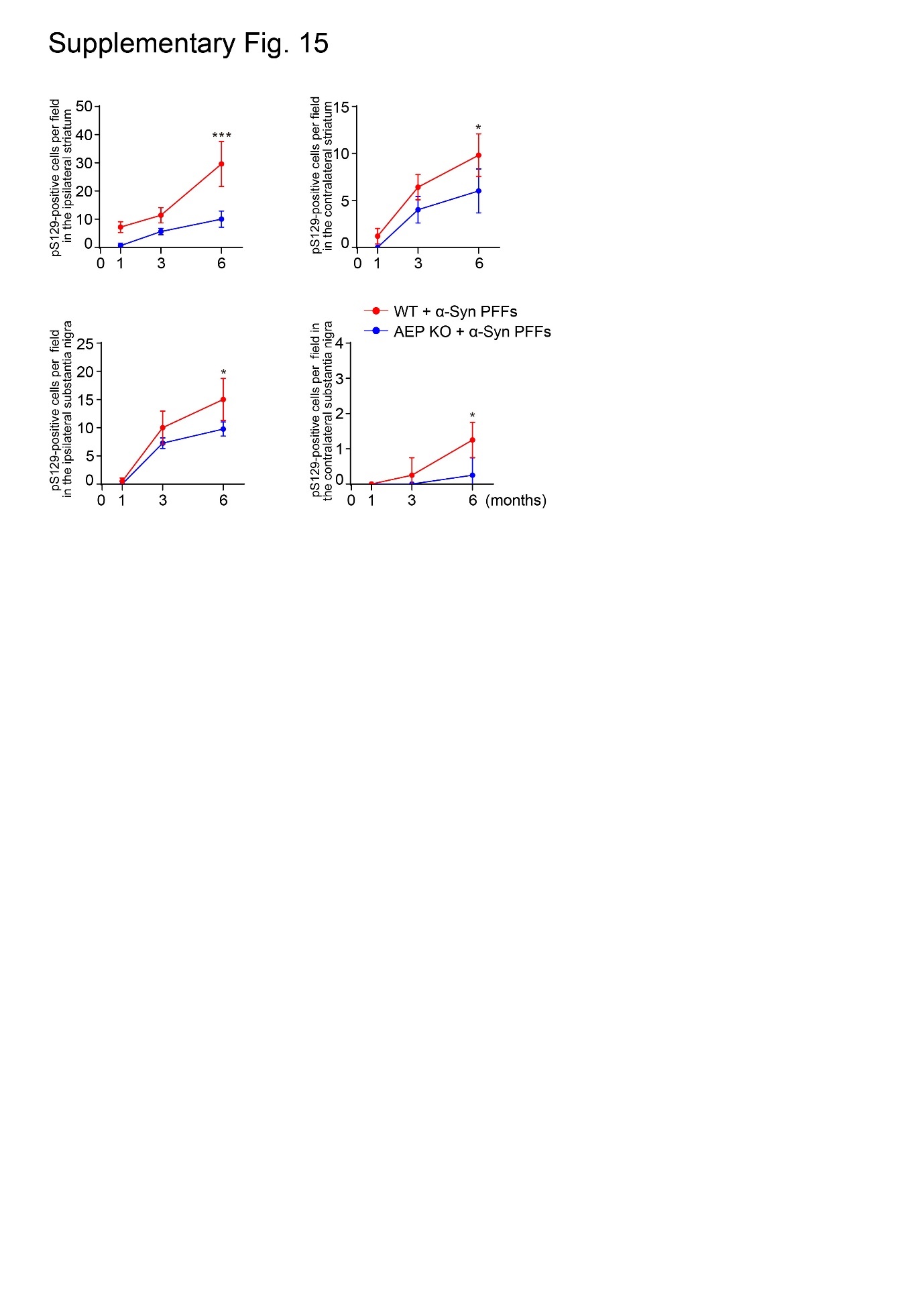


**Supplementary Figure 15.** **Quantification of α-syn pathology in wild-type and AEP KO mice injected with α-syn PFFs. Related to Figure 7.**

The quantification of pS129-positive signals in the striatum and substantia nigra in Figure 7A (mean ± SD; n = 4-5 mice per group; **P* < 0.05; ****P* < 0.001; two-way ANOVA).


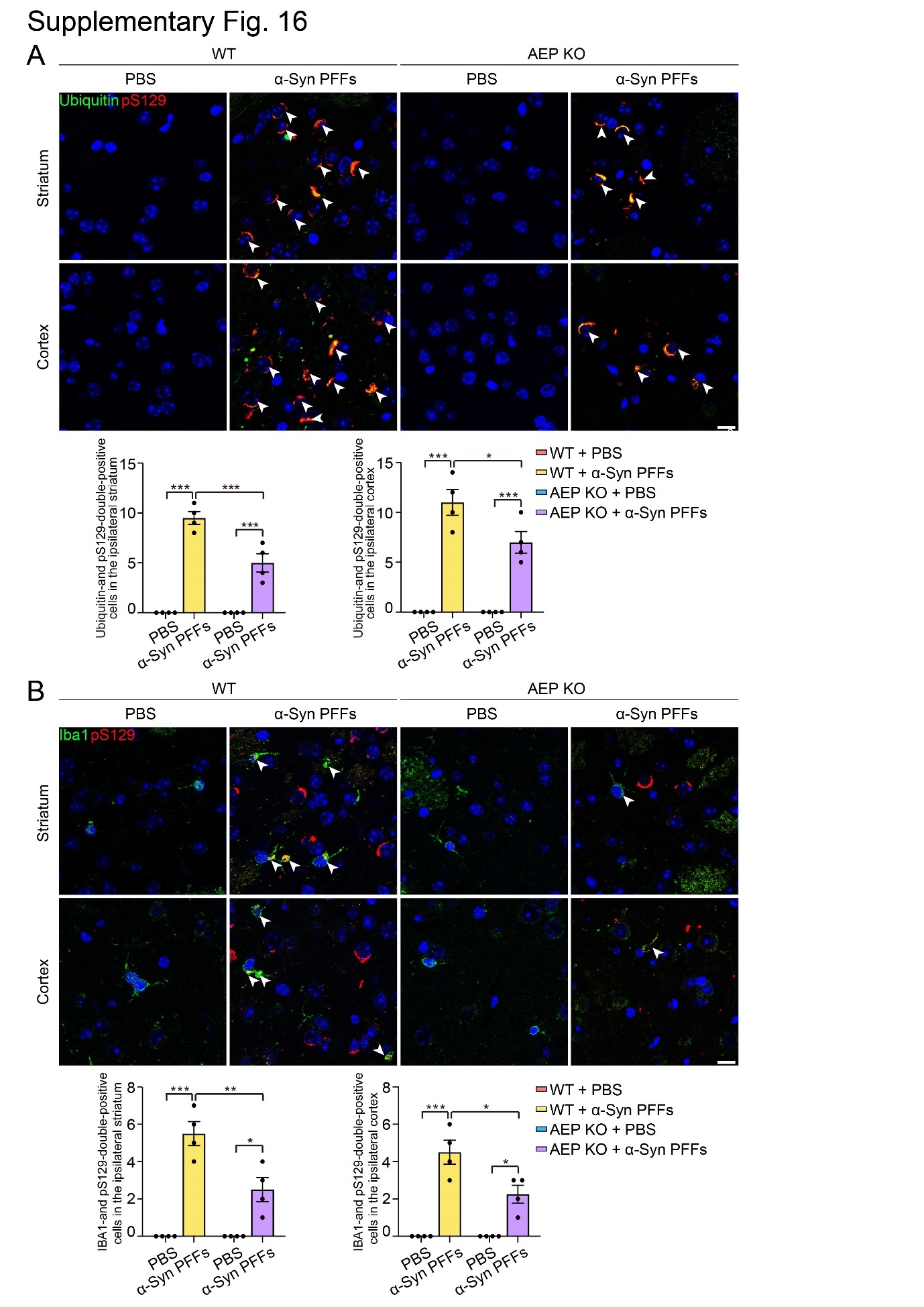


**Supplementary Figure 16.** **Deletion of AEP reduces α-syn pathology *in vivo*. Related to Figure 7.**

(A, B) Immunofluorescence of ubiquitin, Iba1, and pS129 in the striatum and cortex from mice injected with PBS or α-syn PFFs at 6 mpi (mean ± s.e.m.; n = 4 mice per group; **P* < 0.05; ***P* < 0.01; ****P* < 0.001; two-way ANOVA). Ubiquitin/Iba1, green; pS129, red; DAPI, blue. Scale bar, 10 μm.


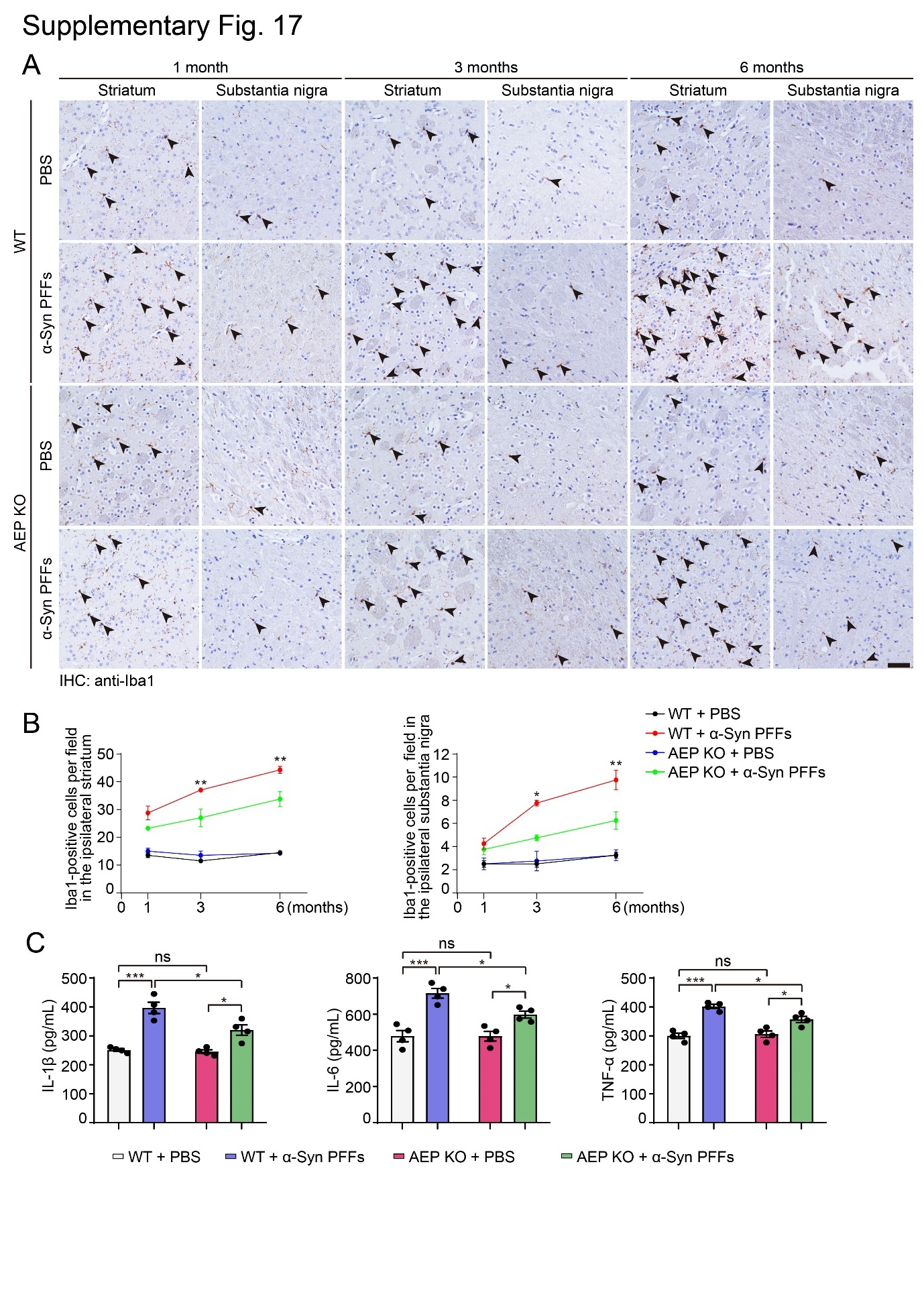


**Supplementary Figure 17.** **The density of microglia in wild-type and AEP KO mice. Related to Figure 7.**

(A) Representative images of microglia in PFF-injected wild-type and AEP KO mice. Scale bar, 50 μm. (B) Quantification of Iba1-positive signals in the ipsilateral striatum and substantia nigra (mean ± s.e.m.; n = 4 mice per group; **P* < 0.05; ***P* < 0.01; two-way ANOVA). (C) ELISA quantification of IL-1β, IL-6, and TNF-α in brain lysates from wild-type or AEP KO mice treated with PBS or α-syn PFFs at 6 mpi (mean ± s.e.m.; n = 4 mice per group; **P* < 0.05; ****P* < 0.001; two-way ANOVA). ns, not significant.

**Table S1.** Mass spectrometry analysis of the membrane proteins that interact with α-syn PFFs.

| **Gene** | **Fibril/Monomer** | **log2 Fibril/Monomer** | **Diff Sig** |
| --- | --- | --- | --- |
| **TREM2** | 539.5610684 | 9.075642445 | + |
| **RPN2** | 174.5085705 | 7.447154082 | + |
| **GPI** | 164.2000215 | 7.359310506 | + |
| **CCDC47** | 105.8627191 | 6.726050805 | + |
| **SNRNP40** | 96.52581729 | 6.592842959 | + |
| **TFG** | 78.91237102 | 6.302179583 | + |
| **NDUFS2** | 69.57435411 | 6.120483705 | + |
| **CPOX** | 64.25898426 | 6.005826271 | + |
| **TMX4** | 63.39290253 | 5.98624942 | + |
| **DNAJC11** | 60.75240459 | 5.924869607 | + |
| **GFPT2** | 58.40042674 | 5.867907006 | + |
| **LPCAT1** | 52.10205895 | 5.70326848 | + |
| **EIF3E** | 48.93426087 | 5.612773003 | + |
| **LAMP2** | 39.15607636 | 5.291164302 | + |
| **STX18** | 36.99413558 | 5.209224683 | + |
| **MTDH** | 30.21700229 | 4.917288638 | + |
| **BCS1L** | 29.32350933 | 4.873985865 | + |
| **FAM98B** | 28.75473435 | 4.845727604 | + |
| **UBA1** | 25.97040853 | 4.698796804 | + |
| **PSMD2** | 25.70654643 | 4.684063898 | + |
| **LNPK** | 23.96277313 | 4.582722971 | + |
| **MAVS** | 23.54984769 | 4.557645824 | + |
| **AUP1** | 23.13802878 | 4.532194056 | + |
| **PEX14** | 22.67282284 | 4.502892117 | + |
| **VRK1** | 22.34894934 | 4.482135105 | + |
| **ILVBL** | 20.84567429 | 4.381676135 | + |
| **FAR1** | 18.20502755 | 4.186265019 | + |
| **CKAP4** | 18.07931113 | 4.176267803 | + |
| **SACM1L** | 17.48148356 | 4.127755719 | + |
| **TMPO** | 16.22481119 | 4.020129784 | + |
| **NUDC** | 15.73160011 | 3.975593514 | + |
| **NCLN** | 15.66721253 | 3.969676617 | + |
| **DHCR24** | 14.69821876 | 3.877569424 | + |
| **POMK** | 14.55444544 | 3.863387965 | + |
| **G3BP1** | 14.35997913 | 3.843981748 | + |
| **ACSF2** | 13.27012897 | 3.730110487 | + |
| **TRABD** | 12.92647141 | 3.692256606 | + |
| **CCDC51** | 11.47091604 | 3.519908701 | + |
| **MPHOSPH10** | 11.4673129 | 3.519455464 | + |
| **SPTLC2** | 11.27718585 | 3.495335192 | + |
| **TMX3** | 9.913025572 | 3.309325452 | + |
| **ATP1B1** | 9.559435173 | 3.256925378 | + |
| **RMDN3** | 9.268931784 | 3.212403082 | + |
| **SLC7A5** | 9.248982634 | 3.209294681 | + |
| **CYP51A1** | 8.62162852 | 3.107960403 | + |
| **CYP20A1** | 8.424072755 | 3.074517896 | + |
| **ARHGAP1** | 8.078018524 | 3.014001454 | + |
| **LETM1** | 7.651384412 | 2.935720807 | + |
| **TIMM17A** | 7.65085238 | 2.935620487 | + |
| **LMAN1** | 7.622910348 | 2.930341909 | + |
| **ENPP4** | 7.223885103 | 2.852774947 | + |
| **STX7** | 7.006477982 | 2.808689412 | + |
| **ALDH6A1** | 7.000068199 | 2.807368978 | + |
| **REEP6** | 6.767959773 | 2.758720993 | + |
| **DSG2** | 6.472788744 | 2.694387419 | + |
| **SGPL1** | 6.404748168 | 2.679141846 | + |
| **ABHD12** | 6.108200837 | 2.610747499 | + |
| **SCCPDH** | 5.497274337 | 2.458716478 | + |
| **TMEM43** | 5.30361654 | 2.40697647 | + |
| **CS** | 5.104781502 | 2.351849211 | + |
| **JAM3** | 4.970695665 | 2.313447776 | + |
| **TMPO** | 4.607164179 | 2.20387901 | + |
| **SEC63** | 4.52420435 | 2.177664095 | + |
| **DHODH** | 4.359946609 | 2.124310468 | + |
| **ATAD1** | 4.22023896 | 2.07732469 | + |
| **MRS2** | 3.812329014 | 1.930672633 | + |
| **NDRG1** | 3.772386734 | 1.915477585 | + |
| **NSDHL** | 3.73515308 | 1.901167371 | + |
| **TTYH3** | 3.678580842 | 1.879149297 | + |
| **FKBP8** | 3.665875555 | 1.874157812 | + |
| **PXMP2** | 3.585661182 | 1.842239171 | + |
| **TMEM201** | 3.585352446 | 1.842114945 | + |
| **FIS1** | 3.541655658 | 1.824423951 | + |
| **PDIA6** | 3.394671726 | 1.763272068 | + |
| **LEMD2** | 3.393560683 | 1.762799811 | + |
| **ALG11** | 3.383696575 | 1.758600204 | + |
| **BCL2L13** | 3.269358063 | 1.709007391 | + |
| **DNAJB12** | 3.113964686 | 1.638752584 | + |
| **AIFM1** | 3.105339163 | 1.634750847 | + |
| **TIMM50** | 3.041278296 | 1.604677838 | + |
| **CXADR** | 2.832291719 | 1.501969867 | + |
| **PERP** | 2.770903348 | 1.470356389 | + |
| **MAN1A1** | 2.752972018 | 1.460989946 | + |
| **CAMK2D** | 2.74455982 | 1.456574784 | + |
| **SSR1** | 2.677929215 | 1.421117827 | + |
| **PREB** | 2.676171394 | 1.420170515 | + |
| **PRRC1** | 2.664326821 | 1.413771062 | + |
| **NUP205** | 2.659422595 | 1.411113047 | + |
| **BPNT2** | 2.530193377 | 1.339247651 | + |
| **NIF3L1** | 2.487840971 | 1.314894268 | + |
| **C6orf89** | 2.344665885 | 1.229382353 | + |
| **LRRC59** | 2.250086458 | 1.169980437 | + |
| **VAPB** | 2.089613474 | 1.063236105 | + |
| **PANX1** | 2.087490882 | 1.061769896 | + |
| **CANX** | 2.04021208 | 1.028719128 | + |
| **CLCC1** | 2.004267343 | 1.003074958 | + |
| **DDOST** | 1.981084403 | 0.986290347 | + |
| **ESYT1** | 1.961146894 | 0.9716976 | + |
| **RPN1** | 1.922899427 | 0.943283308 | + |
| **BSG** | 1.908753316 | 0.932630663 | + |
| **BRI3BP** | 1.826918759 | 0.86941248 | + |
| **ZMPSTE24** | 1.797241542 | 0.845784314 | + |
| **PPFIA1** | 1.79342339 | 0.842716118 | + |
| **GSK3B** | 1.713575581 | 0.777009826 | + |
| **PROCR** | 1.696913243 | 0.762912807 | + |
| **ERGIC2** | 1.685953574 | 0.753564809 | + |
| **CYB5A** | 1.679229801 | 0.747799676 | + |
| **FAF2** | 1.672902593 | 0.742353445 | + |
| **PIGK** | 1.66656488 | 0.736877483 | + |
| **MTX1** | 1.500321604 | 0.585271786 | + |
| **PARVB** | 1.476462479 | 0.562144694 |  |
| **EFNB1** | 1.443426749 | 0.529497895 |  |
| **COX4I1** | 1.414239767 | 0.500026733 |  |
| **TMEM109** | 1.403065431 | 0.48858229 |  |
| **PACC1** | 1.39893364 | 0.484327528 |  |
| **STX5** | 1.394329572 | 0.479571605 |  |
| **DHCR7** | 1.375489761 | 0.4599454 |  |
| **IKBIP** | 1.32978022 | 0.411187823 |  |
| **GDAP1** | 1.297875072 | 0.376151523 |  |
| **TMEM179B** | 1.16446998 | 0.219673447 |  |
| **TMEM65** | 1.163609745 | 0.218607284 |  |
| **CYB5R3** | 1.116325125 | 0.158757267 |  |
| **TOMM70** | 1.067256348 | 0.093906743 |  |
| **BST2** | 1.064504046 | 0.090181433 |  |
| **PTRH2** | 0.981411765 | -0.027069529 |  |
| **APMAP** | 0.967923327 | -0.047035325 |  |
| **KTN1** | 0.967384214 | -0.047839099 |  |
| **METTL2B** | 0.966297445 | -0.049460747 |  |
| **SYPL1** | 0.962104615 | -0.055734321 |  |
| **ALG1** | 0.957026262 | -0.063369581 |  |
| **TMEM70** | 0.882661865 | -0.180067227 |  |
| **M6PR** | 0.862859663 | -0.212802159 |  |
| **ERGIC3** | 0.839965919 | -0.251597302 |  |
| **SPTLC1** | 0.827951236 | -0.272382295 |  |
| **COA1** | 0.822091867 | -0.282628474 |  |
| **ATP1B3** | 0.801868729 | -0.318562016 |  |
| **NDUFA13** | 0.789846252 | -0.340356242 |  |
| **HACD3** | 0.746337742 | -0.422099451 |  |
| **ARMCX3** | 0.671420373 | -0.574711781 |  |
| **REEP5** | 0.647273035 | -0.627553691 | - |
| **ATL3** | 0.634828617 | -0.65556093 | - |
| **RTN4** | 0.62643042 | -0.674773821 | - |
| **PTPN1** | 0.623693286 | -0.681091367 | - |
| **MTCH2** | 0.605156038 | -0.724620909 | - |
| **NDUFA9** | 0.54766214 | -0.868641944 | - |
| **NDUFB11** | 0.522263541 | -0.937150102 | - |
| **SEC11A** | 0.509509642 | -0.972818645 | - |
| **TSPAN3** | 0.469824323 | -1.089806691 | - |
| **SSR4** | 0.464908141 | -1.104982404 | - |
| **SLC25A4** | 0.380005762 | -1.395906801 | - |
| **RRM2B** | 0.37135756 | -1.429119148 | - |
| **GNAS** | 0.334666565 | -1.579203671 | - |
| **RPS10** | 0.331298739 | -1.593795382 | - |
| **MT-CO2** | 0.296776504 | -1.752551216 | - |
| **CYC1** | 0.257494621 | -1.957385799 | - |
| **HMOX2** | 0.249065284 | -2.005404149 | - |
| **CD81** | 0.245122456 | -2.028425439 | - |
| **SLC25A5** | 0.241819436 | -2.047997892 | - |
| **DSC3** | 0.235092134 | -2.08870183 | - |
| **CD9** | 0.216094594 | -2.210265116 | - |
| **PGRMC2** | 0.215197721 | -2.216265294 | - |
| **TMED7** | 0.197840267 | -2.337592003 | - |
| **TMX1** | 0.191725017 | -2.382889495 | - |
| **TFRC** | 0.180678076 | -2.468506636 | - |
| **EMD** | 0.173364119 | -2.528122761 | - |
| **EMC7** | 0.161810727 | -2.627620841 | - |
| **TOMM22** | 0.151245673 | -2.725034227 | - |
| **SFXN1** | 0.148578492 | -2.750702807 | - |
| **MPZL1** | 0.120536274 | -3.052460721 | - |
| **PGRMC1** | 0.113953558 | -3.133482123 | - |
| **CLDN1** | 0.101055312 | -3.306782929 | - |
| **RTN3** | 0.097038604 | -3.365297395 | - |
| **FKBP11** | 0.088154407 | -3.503823488 | - |
| **TMEM126A** | 0.082363773 | -3.601846268 | - |
| **TMED9** | 0.081796034 | -3.611825303 | - |
| **LAMP1** | 0.072169419 | -3.792468543 | - |
| **TMED10** | 0.064239813 | -3.960388491 | - |
| **PEX3** | 0.060585965 | -4.044872556 | - |
| **EMC3** | 0.059836514 | -4.062830065 | - |
| **NCEH1** | 0.053381368 | -4.22751992 | - |
| **CAV1** | 0.046092959 | -4.439309802 | - |
| **SLC25A11** | 0.041124747 | -4.603849389 | - |
| **TIMM23B** | 0.034188662 | -4.870338214 | - |
| **SRPRB** | 0.028540559 | -5.130842577 | - |
| **SLC16A3** | 0.027480354 | -5.185455597 | - |
| **SLC25A6** | 0.026856087 | -5.21860707 | - |
| **SEC22B** | 0.024426944 | -5.355382802 | - |
| **DSG1** | 0.022976855 | -5.443674877 | - |
| **STOM** | 0.014916637 | -6.066933901 | - |
| **HSD17B12** | 0.011600059 | -6.42972399 | - |
| **TTN** | 0.008200707 | -6.930035932 | - |
| **DSC1** | 0.006659506 | -7.23036908 | - |
| **PLP2** | 0.00307584 | -8.344803797 | - |
| **MACF1** | 0.002905465 | -8.427015181 | - |

**References**

[1] C. E. Prada, E. Jousma, T. A. Rizvi, J. Wu, R. S. Dunn, D. A. Mayes, J. A. Cancelas, E. Dombi, M.-O. Kim, B. L. West, G. Bollag, N. Ratner, *Acta Neuropathol* **2013**, *125*, 159.
